# The Cyst-Dividing Bacterium *Ramlibacter tataouinensis* TTB310 Genome Reveals a Well-Stocked Toolbox for Adaptation to a Desert Environment

**DOI:** 10.1371/journal.pone.0023784

**Published:** 2011-09-01

**Authors:** Gilles De Luca, Mohamed Barakat, Philippe Ortet, Sylvain Fochesato, Cécile Jourlin-Castelli, Mireille Ansaldi, Béatrice Py, Gwennaele Fichant, Pedro M. Coutinho, Romé Voulhoux, Olivier Bastien, Eric Maréchal, Bernard Henrissat, Yves Quentin, Philippe Noirot, Alain Filloux, Vincent Méjean, Michael S. DuBow, Frédéric Barras, Valérie Barbe, Jean Weissenbach, Irina Mihalcescu, André Verméglio, Wafa Achouak, Thierry Heulin

**Affiliations:** 1 CEA, Lab Ecol Microbienne Rhizosphere & Environm Extre, iBEB, DSV, Saint-Paul-lez-Durance, France; 2 CNRS, Unite Mixte Rech Biol Vegetale & Microbiol Enviro, UMR 6191, Saint-Paul-lez-Durance, France; 3 Université Aix Marseille, Saint-Paul-lez-Durance, France; 4 Laboratoire de Chimie Bactérienne, UPR-CNRS 9043, Institut de Microbiologie de la Méditerranée, Aix-Marseille Université, Marseille, France; 5 Laboratoire de Microbiologie et Génétique Moléculaire, Université de Toulouse, UPS, Toulouse, France; 6 CNRS, LMGM, Toulouse, France; 7 Architecture et Fonction des Macromolécules Biologiques, UMR 6098, CNRS, Université de la Méditerranée, Marseille, France; 8 Laboratoire d'Ingénierie des Systèmes Macromoléculaires, CNRS-Aix Marseille Université, Institut de Microbiologie de la Méditerranée, Marseille, France; 9 Laboratoire de Physiologie Cellulaire Végétale, CNRS/CEA/INRA/Université Joseph Fourier, CEA-Grenoble, Grenoble, France; 10 INRA, Micalis, Thiverval-Grignon, France; 11 Laboratoire de Génomique et Biodiversité Microbienne des Biofilms, Université Paris-Sud 11, Institut de Génétique et Microbiologie, CNRS UMR 8621, Orsay, France; 12 CEA, DSV, IG, Genoscope, Evry, France; 13 Université Grenoble 1/Centre National de la Recherche Scientifique, Laboratoire Interdisciplinaire de Physique, Unité Mixte de Recherche 5588, Grenoble, France; Universidad Miguel Hernandez, Spain

## Abstract

*Ramlibacter tataouinensis* TTB310^T^ (strain TTB310), a betaproteobacterium isolated from a semi-arid region of South Tunisia (Tataouine), is characterized by the presence of both spherical and rod-shaped cells in pure culture. Cell division of strain TTB310 occurs by the binary fission of spherical “cyst-like” cells (“*cyst-cyst*” division). The rod-shaped cells formed at the periphery of a colony (consisting mainly of cysts) are highly motile and colonize a new environment, where they form a new colony by reversion to cyst-like cells. This unique cell cycle of strain TTB310, with desiccation tolerant cyst-like cells capable of division and desiccation sensitive motile rods capable of dissemination, appears to be a novel adaptation for life in a hot and dry desert environment. In order to gain insights into strain TTB310's underlying genetic repertoire and possible mechanisms responsible for its unusual lifestyle, the genome of strain TTB310 was completely sequenced and subsequently annotated. The complete genome consists of a single circular chromosome of 4,070,194 bp with an average G+C content of 70.0%, the highest among the *Betaproteobacteria* sequenced to date, with total of 3,899 predicted coding sequences covering 92% of the genome. We found that strain TTB310 has developed a highly complex network of two-component systems, which may utilize responses to light and perhaps a rudimentary circadian hourglass to anticipate water availability at the dew time in the middle/end of the desert winter nights and thus direct the growth window to cyclic water availability times. Other interesting features of the strain TTB310 genome that appear to be important for desiccation tolerance, including intermediary metabolism compounds such as trehalose or polyhydroxyalkanoate, and signal transduction pathways, are presented and discussed.

## Introduction

*Ramlibacter tataouinensis* TTB310^T^ (strain TTB310) is a betaproteobacterium isolated from sand particles coated on a meteorite fragment buried in a sandy soil of a semi-arid region of South Tunisia (Tataouine). Scanning electron microscopy observations of the weathered meteorite fragments reveal, in addition to alteration zones at the surface of the meteorite crystals (pyroxene and chromite) and secondary calcite crystals resulting from terrestrial weathering [1], [2], the presence of bacterial rods with an unusually small diameter. The strain TTB310 was isolated among a large diversity of bacterial strains based on its cell diameter as the main criterion for the selection, and secondly on its ability to cause the weathering of orthopyroxene. This strain was characterized by the presence of a pleomorphic form [3] with motile rod-shaped (diameter 240 nm) and spherical cells (diameter 800 nm). It was later identified as a new genus and species, *Ramlibacter tataouinensis* [4]. TTB310 is the type strain of this species. One of the most unusual characteristic of strain TTB310 is the coexistence of both spherical and rod-shaped cells [4]–[6]: these features reveal an original cell cycle that likely constitutes the main adaptation of this bacterium to this desert environment, characterized by cycles of air-drying and rehydratation events and long-term desiccation.

**Figure 1:**
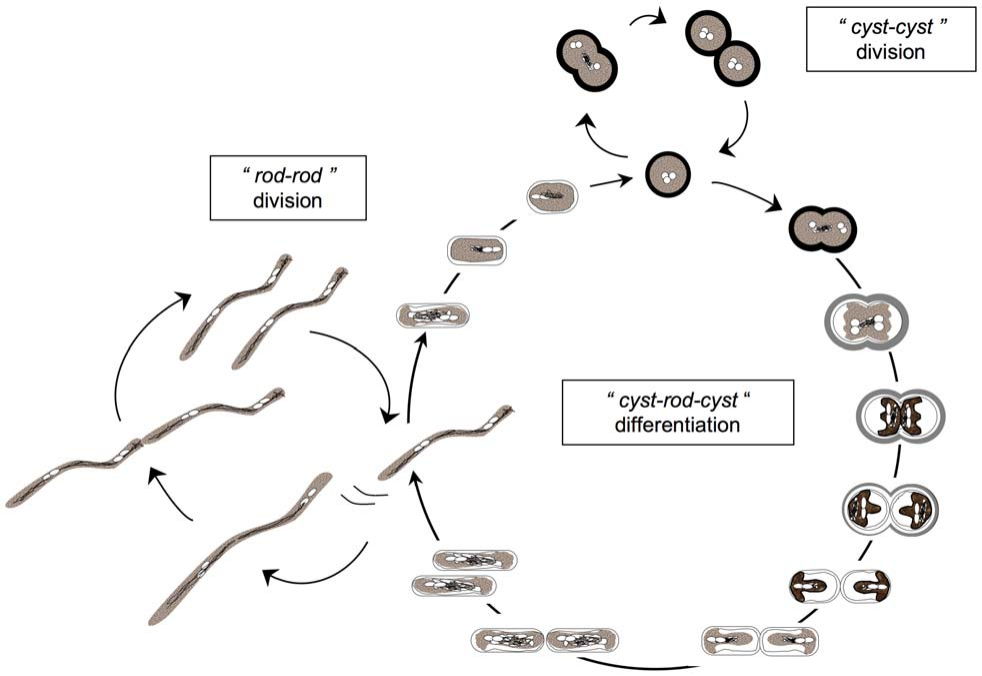
Modelling of *Ramlibacter tataouinensis* TTB310 cell cycle on nutritive agar based on optical and transmission electronic microscopy (TEM). The life cycle includes the “cyst-to-cyst” division step (“*cyst-cyst*” division) and the “cyst-to-rod” division step plus the reversion from “rod-to-cyst” component of the cycle (“*cyst-rod-cyst*” differentiation). The “rod-to-rod” division step (“*rod-rod*” division, Video S1 and Text S1) was included as a step of the “*cyst-rod-cyst*” differentiation. It should be noted that the complex “cyst-to-rod” division step (Video S1; [4]; see [6] for details) occurs at the periphery of the colony [5]. The extracellular polymeric substances (EPS) lysis and cytoplasmic modifications during “cyst-to-rod” division step was depicted according to TEM, which showed that, prior to division and transition into rods, cysts contained condensed cytoplasmic material. These results suggested that the morphological transition occurs solely by the reshaping of cells [6].

The strain TTB310 spherical cells present traits similar to *Azotobacter* cysts, such as the absence of motility, cells embedded within thick extracellular polymeric substances (EPS), the presence of polyhydroxyalkanoate granules in the cytoplasm and a long-term resistance to desiccation [4]. Contrary to cysts of *Azotobacter*, for which the differentiation into rods is necessary for cell division, cell division of strain TTB310 occurs under its “protected” form (cyst), when water and nutrients are available. We thus proposed that spherical cells should be considered “cysts” due to their desiccation tolerance, even if they are not resting cells [4]. This binary fission of spherical “cyst-like” cells (“*cyst-cyst*” division) in an embedded EPS is the basic mechanism by which a bacterial colony grows on solid surfaces and probably an important trait related to its adaptation to desiccation [4], [5]. The rod-shaped cells formed at the periphery of a colony (consisting mainly of cysts) are highly motile (0.1 µm/min), and colonize a new environment, where they form a new colony by reversion to cyst-like cells (“*cyst-rod-cyst*” differentiation) [5], [6]. The formation of the rod-shaped bacteria requires lysis of the EPS, reshaping of the cyst cell including a condensation of cytoplasmic material, and synthesis of a motility apparatus. Conversely, the “rod-to-cyst” transition requires the reshaping of a rod and the synthesis of a new EPS. This original cell cycle of strain TTB310 with desiccation tolerant cyst-like cells capable of division and desiccation sensitive motile rods capable of dissemination seems to be well suited for life in a hot and dry desert and is summarized in Fig. 1.

In order to gain insights into strain TTB310's underlying genetic repertoire and possible mechanisms responsible for its unusual lifestyle, we sequenced the genome of strain TTB310. DNA sequence annotation, using both bioinformatics and manual re-examination by experts in various microbiology fields, shows that strain TTB310 has classical and specific mechanisms for adaptation to desert life, combining both enzymatic and mechanical protective schemes. Both for environmental sensing and for cell cycle control, genomic data suggest that strain TTB310 has developed a highly complex network of two-component systems, which seems to implicate light and perhaps a rudimentary circadian hourglass.

## Results and Discussion

### General features on the genome sequences and structure

The complete genome consists of a single circular chromosome of 4,070,194 bp with an average G+C content of 70.0%, which is the highest of the *Betaproteobacteria* sequenced to date (Table 1). A total of 3,899 predicted coding sequences (CDS), covering 92% of the genome, were identified. Among these, 72% are proteins with a function assigned on the basis of their similarity to other known proteins, 19% are conserved hypothetical proteins and 9% did not display any significant similarity to proteins identified in other organisms. In addition to protein-encoding genes, a single copy of the ribosomal (rRNA) operon, 43 transfer RNAs (tRNA) genes representing all amino acids, and 10 non-coding RNAs were identified. Genome comparisons showed that the strain TTB310 genome was highly similar with those of the *Betaproteobacteria* such as *Polaromonas* sp. JS666, *Delftia acidovorans* SPH-1 and *Acidovorax avenae* subsp. *citrulli* AAC00-1 sharing with them 66, 60 and 60% of its encoded proteins, respectively (Fig. S1).

**Table 1:** General features of the *Ramlibacter tataouinensis* TTB310 genome.

Size (bp)	4,070,194
G+C content (%)	70.0
Coding sequences (CDS)	3899
Coding density (%)	92
Average gene length (bp)	964
Proteins with assigned function	2812 (72%)
Conserved hypothetical proteins	726 (19%)
Hypothetical proteins	361 (9%)
rRNAs	1×(16S-23S-5S)
tRNAs	43
Non-coding RNAs	10

### Carbohydrate metabolism

As expected, strain TTB310 presents the genetic characteristics of an aerobic, chemo-organotrophic bacterial strain [4] (see Table S1 for details). Considering the oligotrophic character of deserts (organic matter <1 mg/g; [7]), we explored the carbon metabolism of strain TTB310 with particular attention. Acetate is used as a carbon and energy source by strain TTB310 [4]. We found in strain TTB310 genes encoding the enzymes catalyzing the transformation of acetate into acetyl-CoA (acetate-CoA ligase, *Rta_15940*), and the first enzymes of the autotrophic dicarboxylate/hydroxybutyrate pathway [8] (Table S1). The first steps of this pathway, from acetate (C2) to oxaloacetate (C4), allow the incorporation of two molecules of CO_2_.

Propionate and β-hydroxybutyrate are also used as carbon and energy sources by this bacterium [4]: propionate can generate acetyl-CoA with propionyl-CoA as an intermediate (propanoate metabolism), and β-hydroxybutyrate can generate acetoacetate (β-hydroxybutyrate dehydrogenase, *Rta_17330*). These three organic acids (acetate, propionate and β-hydroxybutyrate) are well-known carbon substrates for the biosynthesis of polyhydroxyalkanoate (PHA), representing the carbon and energy storage of strain TTB310 [4]. Key enzymes for PHA biosynthesis (PHA polymerase, *Rta_18090*) and catabolism (PHA depolymerase, *Rta_29420*) are present. The pentose phosphate pathway is complete, along with that for pyruvate metabolism. The citric acid cycle (TCA cycle) is also complete and associated to the glyoxylate bypass (malate synthase, *Rta_02700*; isocitrate lyase, *Rta_23660*). In the glyoxylate cycle, oxaloacetate (C4) can be regenerated from phosphoenol-pyruvate (C3) with PEP-carboxylase with the fixation of one CO_2_ (*Rta_28690*). All genes necessary for glycolysis or gluconeogenesis (from α-D-glucose and β-D-glucose to pyruvate) are present, but glucose assimilation was not detected in strain TTB310 [4]: this is probably due to the absence of glucose transporter. Among all the transporters, Rta_24150 appears to be the best candidate to import the different carbon sources metabolized by strain TTB310 including acetate, pyruvate, β-hydroxybutyrate, γ-hydroxybutyrate, DL-lactate, and propionate.

### Tolerance to oxidative stress and DNA repair mechanisms: enzymatic protections

We examined the strain TTB310 genome for the presence of genes encoding for proteins involved in defense mechanisms against the toxicity of reactive oxygen species (ROS). strain TTB310 possesses basic but apparently sufficient equipment with one cytoplasmic (*Rta_11320*) and one periplasmic superoxide dismutase (*Rta_21880*) to cope with the presence of superoxide. Concerning peroxide elimination, all various pathways present in organisms such as *Escherichia coli*, *Xanthomonas campestris* and *Saccharomyces cerevisiae* are found in strain TTB310, with some enzymes even found in multiple copies. The genes of strain TTB310 potentially involved in peroxide scavenging pathways are summarized in Fig. S2. The reductase enzymes, such as TrxB and AhpF, are also present. Although the genes encoding one glutathione-synthetase (*Rta_02450*) and five thioredoxins (*Rta_*05290, *Rta_17070*, *Rta_23420*, *Rta_30710*, *Rta_36760*) to complete the pathways are present, genes encoding for a glutathione reductase could not be found. However, four additional genes (*Rta_11850*, *Rta_13470*, *Rta_22660*, *Rta_29620*) similar to *trxB* (*Rta_31670*) and *ahpF* (*Rta_24200*) are present, though whether one of them is a glutathione reductase remains to be determined. Strain TTB310 is therefore equipped to adapt to various peroxide and superoxide stresses with a classical set of enzymes. One can however note the presence of genes encoding for enzymes involved in carotenoid biosynthesis (*Rta_07680* to *Rta_07730*) to quench ROS in the presence of light, in accordance with the presence of carotenoid pigments in strain TTB310 [4].

Strain TTB310 encodes a complete set of enzymes known to be required for DNA replication, DNA recombination, and for various DNA repair mechanisms. Relevant to the strain TTB310 life cycle, three proteins are potentially repairing DNA photo-damage: (i) a candidate deoxyribodipyrimidine photolyase (photoreactivating enzyme) PhrB (*Rta_34120*) highly common in *Betaproteobacteria* and responsible for the repair of UV-induced DNA damages in a blue light dependent manner; (ii) a candidate deoxyribodipyrimidine photolyase (*Rta_37150*), highly similar to the *Rhodobacter sphaeroides* RSP_3077 protein proposed to act DNA photorepair [9]; and (iii) a conserved hypothetical protein (CHP) distantly related to the spore photoproduct lyase protein SplB from *Bacillus subtilis* (*Rta_25110*). In conclusion, both for tolerance to oxidative stresses and DNA repair mechanisms, strain TTB310 seems to use a “classical” set of enzymes to cope with the drastic semi-desertic conditions, including enzymes for carotenoid biosynthesis and for DNA photo-damage repair.

### Carbohydrate-active enzymes: mechanical protections (exopolysaccharide and trehalose synthesis and degradation)

As explained in the introduction and illustrated by the “classical” set of enzymes used for the tolerance to oxidative stresses and DNA repair mechanisms, we hypothesize that the cyst extracellular polymeric substances (EPS), including exopolysaccharides, constitutes the main physical barrier protecting strain TTB310 from dessiccation/rehydratation cycles. A systematic search for genes encoding carbohydrate-active enzymes was thus carried out to corroborate the existence of exopolysaccharide synthesis and degradation proteins in strain TTB310. A total of 25 glycosyl-hydrolases (GHs) and 40 glycosyl-transferases (GTs) could be identified (Table S2), corresponding respectively to 0.65% and 1.0% of the CDSs of the genome. These percentages are in the average range for bacterial and eukaryotic genomes, whether for GHs alone or GTs alone [10]. The genome encodes a number of expected features such as peptidoglycan, osmoregulated periplasmic glucans, lipopolysaccharide and exopolysaccharide biosynthesis pathways (Table S2).

Interestingly, in strain TTB310 all but one of the identified GHs belong to families known to degrade equatorial glycosidic bonds of substrates (e.g. β-linked for a D-*gluco* configuration). The only exception is a gene that encodes a candidate intracellular α,α-trehalase (*Rta_36490*) that belongs to a distinct subfamily of the large glycosidase family GH15 found in an operon-like gene cluster also containing a gene encoding a candidate trehalose 6-phosphate phosphatase (*Rta_36480*) and a α,α-trehalose-6-phosphate synthase (*Rta_36500*). The disaccharide trehalose is widely distributed in nature and can be found in many organisms, including bacteria, fungi, plants, invertebrates and mammals. It has been shown that trehalose can protect proteins and cellular membranes from inactivation or denaturation caused by a variety of stress conditions, including desiccation, dehydration, heat, cold, and oxidation [11]. Trehalose is likely to be an essential component of the metabolism of strain TTB310 since this organism is subjected to all of the above. Many free-living bacteria store carbon in the form of bacterial glycogen. It has been shown that obligate bacterial parasites and symbionts tend to lose their glycogen metabolism [12]. Strain TTB310 is remarkable in that it has no candidate gene involved in glycogen metabolism despite being a free-living bacterium (in strain TTB310, carbon is stored as PHA). Due to the absence of the glycogen pathway, all the pool of glucose in strain TTB310 can be directed towards the trehalose pathway.

The analysis of the stereochemistry of the glycosidic bonds built by the 40 GTs found in the strain TTB310 genome reveals that a majority are involved in the formation of equatorial (eg β-linked for a D-*gluco* configuration) glycosidic bonds, but 12 (from families GT4, GT8 and GT20) are likely to be involved in the formation of axial (eg α-linked for a D-*gluco* configuration) glycosidic bonds. The function of only one of these 13 α-bond building GTs can be confidently assigned, namely the α,α-trehalose-6-phosphate synthase, which is accompanied by its hydrolytic counterpart. This leaves a dozen genes encoding GTs involved in the formation of axial (eg α-linked for a D-*gluco* configuration) glycosidic bonds with no known degrading counterpart. It is conceivable that the glycoconjugate products of some these GTs are a series of α-linked glycolipids, although no simple glycolipid could be detected in glycerolipid analyses, or that the products are simply not recycled.

In strain TTB310, the only α-cleaving GH is a likely intracellular α,α-trehalase and all other candidate GHs belong to families known to cleave β-glycosidic bonds. This suggests that the subset of β-glycosidases that are exported (Table S2) could be secreted and involved in the rapid breakdown of the abundant EPS during the “cyst-to-rod” transition and, by inference, that the EPS is made of mainly β-linked carbohydrates. If the α-bond building glycosyltransferases discussed earlier were involved in the synthesis of the EPS, then its breakdown would be performed by classes of enzymes yet to be discovered (we note that no polysaccharide lyases have been identified in the strain TTB310 genome).

### Membrane glycerolipids: a complex fatty acid biosynthetic system allowing a versatile tuning of membrane fluidity

After the EPS, membranes are the second physical barriers for protecting bacteria from environmental damages. Therefore, the strain TTB310 genome was carefully examined for glycerolipid biosynthesis systems and was completed by a biochemical analysis of inner membrane lipids. Genes for the complete biosynthetic pathway of lipid A derivatives, which characterize the outer membrane of Gram negative bacteria, are present in the strain TTB310 genome, equipping the cell with a robust hydrophobic barrier anchored to the cell wall. For the inner membrane, lipid content analysis reveals that it is characterized by a phospho-glycerolipid profile with little complexity regarding polar heads (Fig. S3). The major phospholipid is phosphatidylethanolamine. No phosphatidylserine could be detected, although two phosphatidylserine synthases were identified in the genome. One fifth of the glycerolipids is phosphatidylcholine, a lipid that is absent from the vast majority of bacteria [13], particularly from *E. coli* or *B. subtilis*, and whose synthesis in strain TTB310 is attributed to a phospholipids-N-methyl transferase (*pmtA*, *Rta_17000*).

Analysis of fatty acid composition by gas chromatography of the acyl methyl esters indicated a striking complexity, with more than 30 molecular species ranging from 14 carbon atoms (C14) to more than 20 carbon atoms (Table S3). The usual straight chain fatty acids (C16 and C18 molecular species) account for half the fatty acids, with a classical profile of saturated (C16∶0, C18∶0) and unsaturated species (C16∶1, C18∶1, C18∶2, C18∶3). The other half comprises even-numbered very-long chain fatty acids (C20, C22, C24), odd-numbered straight chain fatty acids (C15∶0, C17∶0) and branched chain fatty acids (methyl in *iso* and *anteiso* positions). At the genomic level, we detected strain TTB310 genes for fatty acid biosyntheses initiating with a very large set of primers (Fig. S4). In summary, we found that strain TTB310 presents the ability to synthesize even- and odd-numbered, straight and branched chain fatty acids from acetyl-CoA, propionyl-CoA and branched chain amino acid derivatives as starting units. Three key enzymes are involved in the determining steps of these biosyntheses: the branched chain amino acid transaminase (*bcaT*, *Rta_01870*), the α-keto acid dehydrogenase (*bkd*) cluster (*bkdA1*, *Rta_10480*; *bkadA2*, *Rta_10490*; *bkdB*, *Rta_10500*; *lpd*, *Rta_10510*) and the β-ketoacyl-ACP synthase III (*fabH*) (possibly *fabH*-like1 (*Rta_04890*) and *fabH*-like2 (*Rta_04120*)). This complex fatty acid biosynthesis system therefore provides strain TTB310 with both means by which membranes can adjust their fluidity at the level of acyl-lipids: (i) addition of unsaturations and (ii) addition of methyl-branches. Tuning the derived membrane fluidity is therefore one of the possible determining mechanisms operating in the tolerance to temperature [14] and hygrometry variations, and in the shift between growing, gliding, differentiating, and resisting stages.

### Transporters: involvement in osmotic stress tolerance and cell cycle

A detailed study of the strain TTB310 transporters has been carried out. In summary, compared to other betaproteobacterial genomes, the relative transport capability of strain TTB310 and its percentage of importers (∼70%) are similar to those of *Ralstonia solanacearum* and to what is observed in *Burkholderiaceae* species. Due to possible sudden and drastic fluctuation in osmolarity (osmotic stress) encountered in the Saharan environment, special attention has been dedicated to transporters involved in these mechanisms, including: transporters responsible for the fast uptake of potassium (or, less frequently, sodium) to increase the internal osmolarity in response to a hyper-osmotic shock (reviewed in [15]), ABC systems of the *opu* subfamily involved in the uptake of less harmful solutes for the subsequent replacement of K^+^, mechanosensitive (MS) channels implicated in response to hypo-osmotic stress and major intrinsic proteins (MIP) channels involved in passive transport of water and small solutes such as glycerol and urea [16].

The strain TTB310 genome encodes genes similar (*Rta_02420*, *Rta_26850* and *Rta_05740*) to *E. coli* low (Kup or TrkD) and high (TrkH) efficiency K^+^ transporters. The presence of several K^+^ uptake systems might be due to different pH requirements, since TrkA mainly functions at an alkaline pH and Kup at a low pH in *E. coli* [17]. However, analysis of the strain TTB310 genome did not reveal ABC systems of the *opu* subfamily involved in histidine, proline, proline betaine, glycine betaine and choline uptake nor homologs of the betaine/carnitine/choline transporter (BCCT) family of betaine transporters. This class of compatible solutes, very common in rich soil, may be absent in the cell environment of strain TTB310. In such a case, bacteria can respond to the osmotic up shift by synthesizing glutamine, proline and trehalose. The trehalose-centered metabolism of strain TTB310 reported above suggests that this sugar may be used as a compatible solute.

We identified two candidates (*Rta_25200* and *Rta_26800*) and one putative (*Rta_15000*) mechanosensitive-encoding gene, but all of them belong to the small mechanosensitive ion channels (MscS) family.

Finally, a member of the major intrinsic proteins (MIP) family has been predicted (*Rta_23560*). The best-characterized MIP in bacteria (AqpZ from *E. coli*) is involved in short and long-term osmoregulation, exponential growth and bacterial virulence [18]. It mediates the rapid entry or release of water from the cell in response to sudden shifts in extracellular osmolarity. *Rta_23560* likely plays a similar role in strain TTB310 and might be involved in the “water” loss of two-thirds of the cell volume during cyst-to-rod differentiation.

### Protein export and secretion systems: involvement in EPS hydrolysis and cell motility

**Figure 2:**
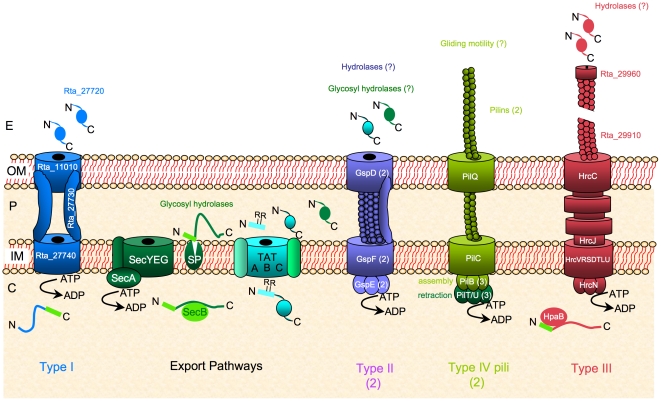
Schematic representation of envelope transport systems in *Ramlibacter tataouinensis* TTB310. In addition to general export pathway (Sec and Tat systems), the strain TTB310 genome encodes one type I secretion system potentially involved in secretion of a large protein, which is a putative adhesin (*Rta_27720*) as found in *Pseudomonas fluorescens* [76], and two type II secretion systems (T2SS) potentially involved in secretion of putative hydrolase implicated in EPS remodelling. The T2SSs clusters (*gspFGHIJKLMCDE* or *gspDHEFG*), each encodes an ATPase (GspE), a secretin (GspD) and a major pseudopilin (GspG), though they contain only one copy of the *gspAB* genes. One type IV pili machinery with different pilins and three PilB paralogs is present in strain TTB310 and is potentially involved in gliding motility. There is only one gene (*pilD*/*gspO*) encoding a prepilin peptidase involved in the maturation of both type II secretion system and type IV pili machinery. The strain TTB310 type III secretion system (T3SS) may be an additional example of the presence of T3SS genes in a nonpathogenic bacterium [29]. It could be involved in the secretion of chitinases through the thick extracellular polymeric substances (EPS) of cyst-cells.

Genes encoding the general inner membrane export system (Sec; [19]), the outer membrane protein insertion system (Bam/Omp85; [20]), the lipoprotein transport system (Lol; [21]) and the non-essential Twin Arginine Translocation (Tat; [22]) system, involved in the transport of folded proteins across the inner membrane, are present in strain TTB310 (Fig. 2, see Table S4 for details including predicted Tat substrates and lipoproteins). Moreover, at least one type II secretion system (T2SS) [23], [24] was found in strain TTB310 (Fig. 2, Table S4), which may be involved in the release of hydrolases required for the breakdown of the exopolysaccharide during the transition from cyst to rod-shaped cells. Indeed, a subset of predicted β-glycosidases displays typical N-terminal signal peptide (Table S2). This is a hallmark for T2SS substrates, which are first translocated in a Sec- or Tat-dependent manner across the inner membane [25]. Once the EPS is degraded, the rod-shaped cells can move in the environment. The strain TTB310 genome analysis indicates that the motility of rod-shaped cells is not supported by flagellar genes (absent in strain TTB310), but probably requires at least type IV pili, since all genes required for assembly of these appendages were found (Fig. 2, Table S4). These data corroborate previous analyses indicating that gliding, which may require type IV pili, is the preferred motility style observed in strain TTB310 [4]–[6]. Furthermore, two genes encoding histidine kinases (*Rta_19330* and *Rta_34130*), similar to CheA and related to genes encoding the FrzE and ChpA proteins involved in gliding and twitching mobility in *Myxococcus xanthus* and *Pseudomonas aeruginosa*, respectively, are present in strain TTB310. We thus propose that the chemotaxis systems in this bacterium may be dedicated to gliding motility [5], [26].

Surprisingly, strain TTB310 possesses one additional gene cluster localized between *Rta_29650* and *Rta_29970*. This cluster encodes proteins highly similar to the plant-pathogen type III secretion system (T3SS) of *Acidovorax avenae* subsp. *citrulli* AAC00-1 (T3SS, Hrp2 family: Fig. 2, Table S4), which is involved in pathogenicity in cucurbits [27]. It should be noted that the strain TTB310 genome encodes two enzymes distantly related to chitinases (*Rta_26180*, *Rta_33120*) and that one of the gene encoding a candidate chitinase (*Rta_29730*) is localized in the middle of the T3SS cluster. Within the vicinity of this cluster, additional genes (*Rta_29974* to *Rta_30025*, Table S4) encode proteins similar to those involved in the last steps of chitinolysis, and in the transport of chitodextrin across the inner membrane in *Collimonas fungivorans* Ter331 [28]. All these genes could confer to strain TTB310 the ability to metabolize extracellular poly-N-acetylglucosamine that could result from a direct biosynthesis by strain TTB310 (*Rta_32250*: related to poly-β-1,6-N-acetylglucosamine synthase) or from other β-N-acetylglucosamine-containing saccharides present in the soil. T3SS is often described as specific to pathogenic bacteria, but has been also found in nonpathogenic bacteria [29]. In strain TTB310, we hypothesize that this T3SS may be used to secrete proteins (for instance chitinases) across the thick EPS of cysts.

### Cell division and cell shape differentiation

According to the bacterial cell morphologies reviewed by Margolin [30], strain TTB310 is an original case due to its transformation from cyst to rod and *vice versa* (Fig. 1). To perform these shape differentiations, the strain TTB310 genome displays highly conserved gene sets required for the cell division and cell shape determination of rod-shaped *Proteobacteria*, including *mreBCD* (*Rta_03840*, *Rta_03830*, *Rta_03820*), *rodA* (*Rta_09910*), *rodZ* (*Rta_18930*), two genes encoding BolA-related proteins (*Rta_08200*, *Rta_20200*) and several penicillin-binding proteins, including the sidewall elongation penicillin-binding protein 2 (*mrdA*: *Rta_03810*). These genetic data and our observations (Fig. 1, Video S1 and Text S1 for details) predict that cylindrical strain TTB310 rod cells grow mainly by extending the length of the cylinder (MreB-dependent sidewall elongation), and that new cell poles are synthesized at cell division (FtsZ-dependent septum formation plus constriction) as observed in *E. coli* [31]. In contrast, strain TTB310 cysts grow *via* their division septa (FtsZ-dependent septum formation) in a manner similar to *Streptococcus pneumoniae* ovococci, as some length extension might still occur [4]. The other shape transitions do not correspond to known models. However, we observed that “cyst-to-rod” differentiation begins by an “ovococcal” division (FtsZ-dependent), followed by the EPS lysis (Fig. 1). After this step, the morphological transition occurs by reshaping of cells (conservation of membrane surface), associated with loss of two-thirds of the cell volume [6] and leads to a rearrangement of the peptidoglycan from a spherical to a rod form, as seen in Video S1. For the “rod-to-cyst” differentiation, a reverse mechanism could be possible with a rearrangement of the peptidoglycan from a rod to a spherical form, associated with the synthesis of a new EPS. It seems that the ability of strain TTB310 to transform its shape from cyst to rod and *vice versa* uses a “classical” set and organization of cell division genes. However, the regulation of the strain TTB310 cell cycle must be tightly controlled, possibly at the transcriptional (sigma factors, transcription regulators of one or two component systems) and post-transcriptional (some His-Asp phosphorelay systems) levels.

### A sophisticated system of signal transduction and light perception: a key for adaptation to extreme environment?

#### DNA-binding proteins

To adjust its adaptive response to environmental changes, the strain TTB310 genome encodes 226 DNA-binding proteins which are, for the most part, Helix-Turn-Helix (HTH) domain-containing proteins: 12 sigma factors, 187 one-component system proteins with HTH (181) or other (6) domains («classical» transcriptional regulators: 4.8% of the genes), and 27 two-component system proteins with HTH domains («transcriptional» response regulators) (http://www.p2tf.org/page.php?base=RamtaDB; Fig. S5). The global number of HTH domain-containing proteins in strain TTB310 is relatively high (220: 12+181+27), and reflects the situation found in a wide diversity of genomes of free-living bacteria in which the one-component systems (here 181) are the main contributors to the total number of the HTH domain-containing proteins [31], [32]. As found in a number of phylogenetically distant free-living bacteria, strain TTB310 exhibits an expansion of the LysR family (27% of the one-component transcription factors) known to be involved in the sensing of a wide range of small molecule ligands [33].

#### Signal transduction: His-Asp phosphorelays

**Figure 3:**
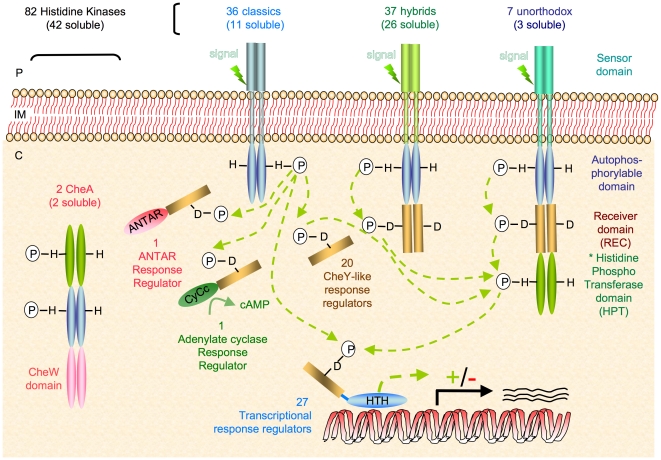
Schematic representation of His-Asp phosphorelays in *Ramlibacter tataouinensis* TTB310. Note: (i) a convergent signalling network due to the higher proportion of histidines kinases (HKs) (82) versus response regulators (RRs) (49); (ii) an intracellular network of signal transduction since half of the HKs (42) are soluble and appear to detect intracellular signals, (iii) the involvement of many two-component system (TCS) (20 CheY-like RRs) in post-transcriptional regulation that likely allow a more rapid adaptation compared to transcriptional regulation (light green dotted arrows indicate possible phosphorylations), and (iv) two chemotaxis systems dedicated to a form of gliding motility.

In addition, strain TTB310 exhibits sophisticated systems involved in adaptive responses to changes in environmental conditions [34]. Indeed, a systematic search for two-component system (TCS) proteins using P2CS (http://www.p2cs.org/page.php?base=RamtaDB) [35], [36] and a manual search, allowed us to identify 131 CDSs potentially involved in TCS or His-Asp phosphorelay signalling in strain TTB310. These systems were classically described as the association of two proteins that communicate through a His-Asp phosphorelay, a histidine kinase sensor protein capable of autophosphorylation on a conserved His residue that can transfer the phosphoryl group to the receiver (REC) domain of a response regulator on a conserved Asp residue (Fig. 3) [37]. TCSs account for about 5.5% of the coding region of the strain TTB310 genome, and represent 3.4% of total protein. This proportion is remarkably elevated, reflecting an important role of TCSs in this bacterium, whereas other signal transduction families [34] were almost missing (Table S5). In strain TTB310, among the 131 CDSs predicted to encode TCS proteins, 82 of them encode histidine kinase sensors (HKs) and 49 encode response regulators (RRs), corresponding to about two sensors per regulator. This unusual ratio between HKs versus RRs suggests a convergent signalling network in this strain, in addition to “classical” two component systems (a HK and its cognate RR) also present.

Interestingly, more than half of the HK predicted proteins are hybrid, since they contain at least a REC domain in addition to the classical HisKA kinase domain (Fig. 3). This is also an unusual situation that probably reflects a particular mode of signal transduction in this organism.

We noted a relative low number of transcriptional regulators (27) in the strain TTB310 genome, which represent only 55% of the 49 predicted RRs, against 80 to 90% usually found (Fig. 3). The fact that about half of the predicted RRs are not transcriptional regulators suggests that TCS outputs could involve protein-protein interactions. These interactions might directly modulate the activity of the RR interacting proteins, and therefore allow a rapid adaptation to environmental changes.

Another particularity of TCSs in strain TTB310 concerns signal detection by HKs. Indeed, more than half of the predicted HKs (42) contain no transmembrane segment (TM), and are therefore predicted to be unable to detect any extracellular signal directly. This observation can be correlated with the elevated number of PAS (59) and PAC (50) domains, described as metabolism related intracellular sensors [38] that are found in 27 soluble HKs and 13 membrane-bound HKs. Among the soluble HKs devoid of PAS/PAC domains, 12 contain a GAF domain, and 2 are associated to a bacteriophytochrome domain. These observations indicate that signal detection in strain TTB310 may occur mostly inside of the cell through PAS and PAC domains.

Regarding TCS signal transduction in strain TTB310, our observations suggest: (i) a convergent signalling network due to the higher proportion of HKs versus RRs; (ii) an intracellular network of signal transduction, since half of the HKs seem to detect intracellular signals, (iii) the involvement of many TCS in post-transcriptional regulation that likely allow a more rapid adaptation compared to transcriptional regulation and (iv), as explained in the secretion system part, two chemotaxis systems dedicated to a form of gliding motility. As found in *Caulobacter crescentus* [39] and suggested in the cyanobacterium *Nostoc punctiforme* (*[Anabaena]* sp. strain PCC 7120) [40] that both possess a complex program of cell differentiation, a part of these systems could be dedicated to the control of the strain TTB310 cell cycle.

#### Light sensing: two red/infrared and four blue-light potential photoreceptors

Strain TTB310 presents one of the higher proportions of light sensing proteins exhibited by a chemotrophic non-phototrophic bacterium [41]. Indeed, six genes encoding potential light sensors that contain all the hallmarks of a bacteriophytochrome [42], a phototropin [43] or a blue light using flavin adenine dinucleotide (BLUF) protein [44] have been identified in strain TTB310: two red/infrared light sensing histidines kinases or bacteriophytochrome photoreceptors (*Rta_25470* and *Rta_28950*), a blue-light sensing histidine kinase or phototropin (*Rta_12790*), and three sensors of blue-light corresponding to BLUF proteins (*Rta_31060*, *Rta_20590*, *Rta_26080*). These proteins may allow strain TTB310 to sense red/infrared (650–750 nm) and blue-light (350–450 nm), which could be an essential feature for adaptation to desert conditions. Indeed, due to the strong correlation existing between light, heat and desiccation in a desert environment, light should be one of the more important external cues allowing strain TTB310 to anticipate desiccation events by induction of protective mechanisms such as rod encystment. In agreement with this hypothesis, preliminary experiments using day/night cycles with continuous light provided by a cool white fluorescent lamp (SYLVANIA GRO-LUX®, 140 µE m^−2^ s^−1^) shows that strain TTB310 growth is greatly reduced during the light period. This phenomenon is associated with a morphological change of rod-shaped cells, which seem to be transformed into the more resistant cyst-like cells that quickly become dominant after light exposure. Due to the emission properties of this fluorescent lamp, which contains little red or far-red light, this suggests that some of the blue-light receptors described above could be involved in rod-shaped cell to cyst-like cell differentiation. Finally, the two bacteriophytochromes (*Rta_25470*, *Rta_28950*) could be involved in the synthesis of the strain TTB310 carotenoids [4], as demonstrated in *D. radiodurans* [45].

### The Rta_04330 (KaiC ATPase)/Rta_04340 (Histidine Kinase) signalling pathway: an ancestral simple hourglass timing mechanism dedicated to anticipate night/day cycle?

Two genes (*Rta_04330* and *Rta_35460*) encoding proteins similar to *Synechococcus elongatus* PCC7942 KaiC protein (SYNPCC7942_1216: *Syn*KaiC) were found in the strain TTB310 genome (*Rta*KaiC). *Syn*KaiC is the core component of a circadian clock that controls the cyclic expression of almost 30 to 64% of *Synechococcus* genes ([46]; see [47]–[52] for recent reviews). Two other proteins, KaiA and KaiB, are important in the robustness of the *Synechococcus* clock. Indeed, oscillations in the phosphorylation state and more recently in the ATPase activity of KaiC have been proposed as the pacemaker of the circadian clock. These oscillations require the action of KaiA and KaiB, which enhance autokinase and autophosphatase activities of KaiC, respectively. A simplified timing system acting only as a 24 h timer, more like an hourglass than a clock, has been recently demonstrated in *Prochlorococcus* in the absence of KaiA protein [53]. Contrary to *Syn*KaiC, *Pro*KaiC is constitutively phosphorylated when incubated alone, and this activity is not modified by the addition of *Syn*KaiA or KaiB from either species [54], [55]. Although two copies of gene encoding core component of a circadian clock are present, surprisingly, neither *kaiA* nor *kaiB* homologs could be found in strain TTB310. However, based on the biochemical properties of the hourglass mechanism found in *Prochlorococcus*, we hypothesized a possible timing role of *Rta*KaiC, in the absence of both KaiA and KaiB partners.

**Figure 4:**
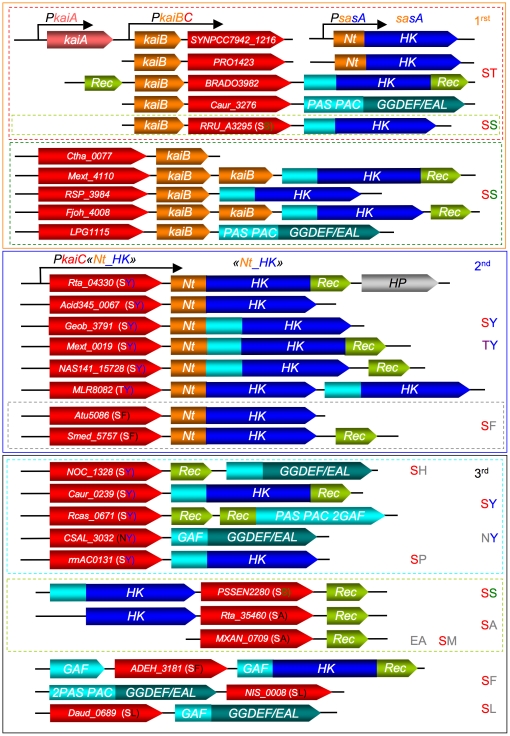
Schematic representation of predicted KaiC genetic organization compared to cyanobacterial KaiABC-SasA «clock system». (SasA is found isolated in *Cyanobacteria* genomes). This representation is based on the phylogeny of predicted KaiC according to TULIP tree (Fig. S6). The first clustering corresponds to colour and *KaiC*-context group name (1^rst^, 2^nd^, 3^rd^) according to the text and exhibits nature of the phosphorylation sites (ST, SS, SY, TY etc…). KaiC proteins (red colour) have been named according to their encoding gene position in database. KaiC neighbouring proteins were represented according to their proteic domain contents: HK, histidine kinase domain constituted of a HisKA and an HATPase_c domains; REC, single domain receiver protein; PAS, PAS domain; PAC, PAC domain; GAF, GAF domain; GGDEF/EAL, GGDEF and EAL domains. For HK, the N-terminal, PAS, PAC or GAF domains have been replaced by blue-light colour (for details see Fig. S6). Nt_HK: HK with a N-terminal «orange» domain exhibiting similarities with cyanobacterial KaiB protein and kaiB-like N-terminal KaiC-interacting sensory HK SasA. Genes are not drawn on scale.

To evaluate this hypothesis, we compared the gene context of *kaiC* in various prokaryotes (Fig. 4). Three *kaiC*-contexts were defined from the phylogenetic tree (Fig. 4 and Fig. S6). In the first *kaiC*-context («orange group»), *kaiC*-*kaiB* genes are clustered. Except for cyanobacteria, almost all show the presence of a histidine kinase or a GGDEF/EAL domains containing protein immediately downstream of a *kaiC* or *kaiB* gene (Fig. 4). A second *kaiC*-context («dark-blue group») shows a simpler and highly conserved organization with *kaiC* followed systematically by a specific histidine kinase (HK) gene and the absence of *kaiB* gene elsewhere in the genome (Fig. 4). A more diverse *kaiC*-context («black group») exhibits a less conserved gene arrangement. The *kaiC* gene is frequently followed by a gene encoding a receiver protein or localized near a signaling protein (HK, GGDEF/EAL domains containing protein) (Fig. 4).

These three *kaiC*-contexts are consistent with the phylogenetic tree of KaiC (Fig. S6). The phosphorylation capacity of KaiC also presents a similar pattern. Indeed, almost all the KaiC proteins of the first and second families exhibit phosphorylable residues at the key positions necessary for their oscillatory activity (Fig. S7). In the third KaiC family («black group»), only one (or none) phosphorylation site conservation is present with the exception of PSEEN2280 (SS profile) or NOC_1328, Caur_0239 and rrnAC0131 (SY profile) (Fig. S6 and S7).

It appears that almost all *kaiC* bacterial genes, with the exception of the cyanobacterial ones, occur near signaling proteins, more frequently upstream of histidine kinase encoding gene. The KaiC homologs unable to phosphorylate two residues, like Rta_35460, probably fail to sustain a cyclic timing mechanism. On the other hand, it is debatable whether KaiC homologs containing two phosphorylation sites, like Rta_04330, could potentially represent an hourglass timing mechanism, even in the absence of KaiB («dark-blue group»). In this family, the *kaiC* gene is systematically followed by histidine kinase (HK) encoding gene (Fig. 4). In the case of strain TTB310, RT_PCR experiments indicate that *Rta_04330* and *Rta_04340* are cotranscribed as part of a single operon (see materiels and methods for details), suggesting that they are partners in the same regulatory pathway.

From the high sequence similarity of the N-terminal sensor domain (denoted *Rta*Nt) of these histidine kinases, we defined a specific consensus sequence. *Rta*Nt exhibits 19% identity (33% similarity, E-value 5e^−05^) with the N-terminal receiver domain of a *Thermosynechococcus elongatus* BP-1 putative two-component response regulator. Moreover, clustal alignment shows that *Rta*Nt exhibits 16% identity (44% similarity) and 10% identity (40% similarity) with the cyanobacterial N-terminal sensor domain of the KaiC-Interacting sensory histidine kinase SasA and KaiB proteins, respectively. These two proteins are known to interact with *Syn*KaiC, with SasA being the key player in the output pathway of the clock «signal». All these observations suggest that these HKs define a highly conserved KaiC-interacting specific sensory HK family, as does SasA protein in cyanobacteria, via the protein-protein interaction module *Rta*Nt.

It is now necessary to demonstrate whether these KaiC «homologs» have a timing function and to search for the cellular processes controlled by this potential rudimentary hourglass, as recently suggested in the heterotrophic bacterium *Pseudomonas putida* [56], and in *Legionella pneumophila* [51]. In the case of strain TTB310, this predicted rudimentary hourglass could be used, in addition to light signals (see above), to anticipate water availability at the dew time in the middle/end of desert nights (winter) and thus direct the growth window to cyclic water availability times.

### Conclusion

The resistance to desiccation of strain TTB310, a bacterium capable of cyst-division, represents a novel adaptation to drastically changing conditions in the desert environment. Strain TTB310 possesses a single circular chromosome of 4,070,194 bp, with the highest G+C content ever observed (70%) for a betaproteobacterial genome, encoding 3,899 predicted proteins (Table 1). DNA sequence annotation, using both bioinformatics and manual re-examination by experts in various molecular microbiology fields, shows that strain TTB310 uses both classical and special toolboxes for adaptation to desert life. Strain TTB310 is only equipped with a classical set of enzymes to adapt to various peroxide and superoxide stresses. However, we note the presence of genes encoding enzymes involved in carotenoid biosynthesis to quench ROS in the presence of light, as expected [4]. In the same way, the strain TTB310 genome encodes a complete set of classical enzymes known to be required for DNA replication, recombination, and for various DNA repair mechanisms, including photo-damage. Besides these “classical” enzymatic protective mechanisms, the EPS may constitute the main physical protection barrier against desiccation/rehydratation cycles in this bacterium. The genome annotation of carbohydrate-active enzymes confirms that an exopolysaccharide synthesis and hydrolysis system is present in strain TTB310, and reveals that desiccation tolerance is probably aided by the biosynthesis of the compatible solute trehalose. Indeed, trehalose protects proteins and membranes from inactivation or denaturation caused by a variety of stresses (e.g. desiccation, heat, oxidation) and is likely to be an essential component of strain TTB310 metabolism, which is subjected to all of the above-mentioned stresses in an arid soil probably devoid of environmental compatible solutes. Secretion system annotation has revealed that strain TTB310 is able to export a subset of ß-glycosidases, potentially secreted and involved in EPS hydrolysis, but also to synthesize type IV pili, that could be implicated in the gliding motility of strain TTB310 and in two chemotaxis systems, as well. While the function of a type III secretion system in a non-pathogenic bacterium needs to be studied, we hypothesize that it could be involved in proteins secretion outside the thick EPS from cysts for nutritional functions (e.g. chitinolysis). Moreover, a complex fatty acid biosynthesis system, with addition of unsaturations or of methyl-branches to acyl-lipids, allows strain TTB310 to adjust membrane fluidity for adapting to temperature and hygrometry variations.

Besides enzymatic and mechanical adaptations, strain TTB310 exibits a highly complex cell cycle well suited for life in hot and dry deserts. As for enzymatic protection, cell division and cell shape determination analysis shows that strain TTB310 uses a “classical” set and organization of cell division genes, with an additional set of peptidoglycan reshaping enzymes. However, the regulation of the strain TTB310 cell cycle must be tightly controlled, likely at the transcriptional (sigma factors, transcription regulators of one or two component systems) and post-transcriptional levels (His-Asp phosphorelay systems). In this context, strain TTB310 exhibits a highly complex network of 131 two-component signal (TCS)-transduction proteins (3.5% of the genes), representing an atypical organization, with convergent signalling networks, as well as an intracellular network for signal transduction, and the involvement of probably more than half of the TCSs in post-transcriptional regulation events that are necessary for rapid adaptation to drastic environmental changes. In summary, strain TTB310 which is the type strain of *R. tataouinensis* possesses all the required systems both for environmental sensing and for cell cycle control. Among them, the occurrence of two HK-bacteriophytochromes, one blue-light sensing HK and three blue light using flavin adenine dinucleotide (BLUF) proteins supports a control of the cell cycle by red/far red and/or blue light. Finally, the presence of a potential rudimentary hourglass is suggested by the presence of a gene encoding a KaiC homologue, followed by an HK. This hourglass could have a timing function, and be used to anticipate water availability at the dew time in the middle/end of the desert winter nights and thus direct the growth window to cyclic water availability times. These features may be a hallmark for adaptation to desert conditions, where exposure to light, high temperature and water deficiency are correlated.

Arid regions are the largest type of terrestrial ecosystem (representing approximately 33% of the terrestrial surface), yet one of the least explored at the level of its biodiversity. This report highlights new adaptation features to desert lifestyle exhibited by this bacterium.

## Materials and Methods

### Cultivation of cells and preparation of genomic DNA

*Ramlibacter tataouinensis* TTB310^T^ (strain TTB310) (described in [4] and available in public strain collection as strain DSM 14655^T^, ATCC BAA-407^T^ or LMG 21543^T^) was cultured in tenfold diluted tryptic soy broth (TSB 1/10, Difco Laboratories). After incubation at 30°C for 72 h with shaking, the cells were harvested for 20 min at 15,000 g and subsequently washed in sterile distilled water. DNA from strain TTB310 was prepared from 200 ml of cultures according to standard procedures [57]. The supernatant fluid was then subjected to a phenol/chloroform extraction and the DNA was recovered after ethanol precipitation.

### Genome Sequencing

The sequencing of the strain TTB310 genome was entirely executed by the Genoscope (Evry, France), using a conventional whole genome shotgun strategy [58]. Four libraries were constructed using different vectors and insert sizes. Three of them were prepared after genomic DNA fragmentation by mechanical shearing. The 3 kb (A, B) and 10 kb (C) fragments were cloned onto pcdna2.1 (A) (INVITROGEN) or pCNS (pSU18 derived) (B, C) vectors. A forth library were obtained using a *Bam*HI partial digest of the genomic DNA and 20 kb inserts were introduced onto pBeloBac11 (D). Vector DNAs were purified and end-sequenced (31202 (A), 21867 (B), 18139 (C) and 6146 (D)) using dye-terminator chemistry on ABI3730 sequencers. A pre-assembly was made without repeat sequences as described by Vallenet *et al.* [59] using Phred/Phrap/Consed software package (www.phrap.org). The finishing step was achieved by primer walking, PCR and *in vitro* transposition technology (Template Generation System™ II Kit; Finnzyme, Espoo, Finland), corresponding to 1525, 219 and 228 additional reads, respectively. The strain TTB310 nucleotide sequence and annotation data have been deposited at GenBank under accession number CP000245 (taxon ID 365046; project ID 35861).

### Gene prediction and annotation

Protein-coding regions in the assembled genome sequence were identified using the gene prediction software FrameD [60] and AmiGene [61]. The results were combined and a search for common genes between the gene identification tools made it possible to eliminate redundancy. All predicted proteins larger than 20 amino acids were analysed for sequence similarity against protein databases (SWISSPROT, TREMBL and PIR). Similarity searches were carried out using BLASTP [62].

Annotation of the complete genome was performed using a bioinformatic tool allowing data management, developed in-house (P. Ortet and M. Barakat, unpublished data). Our tool allows an expert annotation by manual verification and curation of functional protein categories after automatic assignment.

Regions of the genome without CDSs, and CDSs without a database match are re-evaluated by using BLASTX as the initial search, and CDSs are extrapolated from regions of alignment.

Protein functional annotation was based on similarity searches against public databases and domain analysis with HMMER (Sean Eddy http://hmmer.wustl.edu/ 2001).

Functional classification was based on homology searches against the Clusters of Orthologous Groups of proteins (COGs, [63]). rRNA and tRNA genes were identified with BLASTN and tRNA-Scan.

Paralogous families were built as described in Bastien *et al.* [64]. Briefly, a random proteome database of strain TTB310 was built. The longest sequences (>5 kb, 7 sequences) were removed to build up non-redundant proteomes. Each apparent protein of the non-redundant proteome of strain TTB310 was compared to all the sequences of the corresponding random database, using the BLASTP algorithm [62] and the best alignment *P*-values were collected. From the distribution of the self×random *P*-values, a 0.99-percentile was set to define a cutoff. A Z-value was deduced and used as a cutoff value according to the TULIP theorem [65]. Then, the calculated cutoff was used as a criterion to partition the proteome owing to the single-linkage clustering method, using the SW algorithm [66]. We define paralogs as proteins sequences satisfying a Z-value cutoff of 18 and having at lest 30% sequence identity over more than 60% of their lengths.

### Glycerolipids analysis

Glycerolipids have been extracted using organic solvents, and analysed by two-dimensional thin-layer chromatography coupled with methanolysis and gas chromatography, as previously described [67].

### Analysis of the Carbohydrate-Active Enzyme encoding genes

All CDSs were compared, using gapped-BLAST [62] against a library of catalytic and ancillary modules covered by the sequence-based family classification Carbohydrate-Active Enzymes (CAZy at URL: http://www.cazy.org) [68], [69]. The assignment to the various families of glycosidases and transglycosidases (hereafter referred to as glycoside hydrolase or GHs), glycosyltransferases (GTs), polysaccharide lyases (PLs), and ancillary carbohydrate-binding modules (CBMs), provides the foundation to the sequence and mechanism-based annotation of the carbohydrate-active enzyme-encoding genes [70]. This analysis, which integrates the frequent modular structure of this class of enzymes and the polyspecificity of many families, provides an insight into the metabolism of oligo- and polysaccharides by strain TTB310. The list of CDSs assigned to GHs and GTs families is provided in Table S2.

### Analysis of transporter candidates

The annotation of transporter candidates was achieved with the bioinformatic strategy developed for the annotation of ABC transporters [71]. The method has been extended to other transport systems with the annotated transporters retrieved from TransportDB (http://www.membranetransport.org/) [72] and functional annotation was completed with the help of TCDB (http://www.tcdb.org/).

### Cultivation of cells in light/dark cycles

Strain TTB310 was cultured in tenfold diluted tryptic soy broth (TSB 1/10, Difco Laboratories). After incubation at 30°C for 72 h with shaking in the dark, bacteria were spread on TSB 1/10 agar plates (1.5 g l^−1^) and cultured at 30°C in the dark or in light/dark conditions (12 h/12 h) in an incubator equipped with fluorescent lamp (Infors multitron 2). Continuous light was provided by cool white fluorescent lamps (SYLVANIA GRO-LUX®, 140 µE m^−2^ s^−1^). Every hour, a small piece of agar supporting one colony was cut to observe bacteria with a BX50 Olympus microscope equipped with a differential interference contrast (DIC) device and a 100× oil immersion objective (UPlanApo, Olympus) according to [6].

### RNA isolation and RT-PCR

For analysis of *Rta_04330* (1.488 kb) and *Rta_04340* (1.515 kb) gene expression, cells were treated with RNAprotect Bacteria Reagent (Qiagen) prior to RNA isolation using the RNeasy Mini Kit (Qiagen) according to the manufacturer's instructions. RNA samples were treated twice with DNase. For RT-PCR, cDNA was synthesized in 20 µL reactions using 1 µg of RNA and the Transcriptor First Strand cDNA Synthesis Kit (Roche). DNA fragments of 2.5 kb were then amplified in 25 µL reactions using 1 µL of cDNA from the first step, Taq polymerase (Sigma) and two primers designed in *kaiC* gene (*Rta_04330*: Rta04330_Forward GCATCGTGCTCGATTCGCTG) and at the end of the adjacent gene encoding an HK (*Rta_04340*: Rta04340_Reverse GACGAAGTGGAAGTCGAAGCC), respectively. These amplifications were carried out by incubating reactions at 95°C for 5 min prior to 35 cycles of 30 s at 95°C, 30 s at 56°C and 2 min at 72°C, followed by a final step at 72°C for 2 min. Controls for DNA contamination were performed with reactions lacking reverse transcriptase. The amplification of a fragment of 2.5 kb corresponding to 1 kb of *Rta_04330* and to the entire 1.5 kb length of *Rta_04340*, demonstrates that the two genes are co-expressed.

### KaiC phylogenetic analysis

Rta_04330 and Rta_35460 were first aligned with SYNPCC7942_1216 (SynKaiC) with the Basic Local Alignment Search Tool software (BLAST: Align two or more sequences). Other KaiC sequences were retrieved from the non-redundant protein sequences database (nr: NCBI) with Rta_04330 as query and then aligned with the Multiple Sequence Alignment software CLUSTALW (Fig. S7). *Cyanobacteria* and *Proteobacteria* represents the most abundant KaiC containing phylogenetic groups. Therefore, we voluntarily excluded redundant sequences, mostly from *Cyanobacteria* and *Proteobacteria*, for a greater clarity of the representation. Classification of protein sequences was performed with the TULIP 1.1 server (http://malport.bi.up.ac.za/TULIP/index.php) [65], and was based on pairwise alignments and following evolutionary assumptions, according to the TULIP theorem (Theorem of the Upper LImit of a score Probability). Input sequences were compared with the Smith-Waterman method using the following substitution matrix: blosum62.bla. Z-values were estimated after 1000 sequence randomizations. Proteins were classified using a distance matrix derived from Z-value probabilities. The resulting unrooted Tulip tree was drawn with the TreeDyn online software (http://www.phylogeny.fr) [73], [74]. In this case, TULIP tree was consistent with phylogenies described by Dvornyk *et al.* [75] and Loza-Correa *et al*. [51]. The nature of the phosphorylable residues and of the neighbouring genes was added manually on the Fig. S6.

## Supporting Information

Figure S1 **Comparison of the** ***Ramlibacter tataouinensis*** **TTB310 genome against the closest proteobacterial genomes.** Similarity searches were carried out between strain TTB310 and all the complete proteomes present in NCBI database, using BLASTP. The figure was generated with the results of the thirteen most similar genomes (12 betaproteobacteria, 1 alphaproteobacterium). Genomes are represented by successive circles made of coloured sticks representing individual genes. Color code of sticks: orange, strain TTB310 CDS forward; yellow: strain TTB310 CDS reverse; green: similar genes present and found in the same genomic environment in the other genomes (synteny); red: similar genes present in the other genomes. White holes represent an absence of similar genes in the other genomes. Names of the thirteen strains used for genome comparison classified from the inner (most similar) to the outside of the circle: *Polaromonas* sp. JS666, *Delftia acidovorans* SPH-1, *Acidovorax avenae* subsp. *citrulli* AAC00-1, *Polaromonas naphthalenivorans* CJ2, *Acidovorax* sp. JS42, *Leptothrix cholodnii* SP-6, *Methylibium petroleiphilum* PM1, *Rhodoferax ferrireducens* T118, *Azoarcus* sp. BH72, *Ralstonia eutropha* H16 chromosome 1, *Bordetella petrii* DSM 12804, *Burkholderia xenovorans* LB400 chromosome 1, *Bradyrhizobium* sp. ORS278.
(TIFF)

Figure S2 **Genes of** ***Ramlibacter tataouinensis*** **TTB310 potentially involved in peroxide scavenging pathways.**
(TIFF)

Figure S3 **Glycerolipid composition of** ***Ramlibacter tataouinensis*** **TTB310 membranes.** PE, phosphatidylethanolamine, PC, phosphatidylcholine, PG, phosphatidylglycerol, DPG, diphosphatidylglycerol, PI, phosphoinositides. Glycerolipids (100 µg) were resolved by two-dimensional thin layer chromatography (first dimension, chloroform/methanol/water 65∶25∶4; second dimension, chloroform/acetone/methanol/acetic acid/water 100∶40∶20∶10) and visualized after 8-anilino-1-naphthalenesulfonic acid spray.
(TIFF)

Figure S4 **Biosynthesis of even- and odd-numbered, straight and branched chain fatty acids from acetyl-CoA, propionyl-CoA and branched chain amino acids derivatives as starting units in** ***Ramlibacter tataouinensis*** **TTB310.** Determining steps for the distribution of fatty acid molecular species in the final profile include the branched chain amino acid transaminase (*bcaT*), the α-keto acid dehydrogenase (*bkd*) cluster and the β-ketoacyl-ACP synthase III (*fabH*).
(TIFF)

Figure S5 **DNA-binding proteins in** ***Ramlibacter tataouinensis*** **TTB310.** This figure represents the distribution of the transcription factors found in *R. tataouinensis*.
(TIFF)

Figure S6 **Representation of prokaryotic predicted KaiC proteins according to (1) their TULIP tree position, (2) nature of their phosphorylable sites and (3) their genetic organization.** Proteins were classified using a distance matrix derived from Z-value probabilities (see Materials and Methods). We have integrated the RecA protein (Rta_37450, 351 residues) as an outgroup and two archaeal KaiC single domain proteins (SSO1861, 280 residues; SSO2452, 262 residues) recently classified as archaeal RadC and thought to be implicated in DNA repair [77]. (ST) represent the nature of the conserved KaiC phosphorylation sites residues (S, serine; T, threonine; Y, tyrosine; F, phenylalanine; A, alanine; L, leucine; H, histidine; D, aspartic acid). KaiC neighbouring proteins were represented according to their protein domain contents: REC, single domain receiver protein; Nt_PAS_PAC_GAF_HK_REC, hybrid histidine kinase with N-terminal domain composed of a N-terminal region, one PAS, one PAS and one GAF domains; PAS_2PAC_GGDEF_EAL, protein containing one PAS, two PAC, one GGDEF and one EAL domains. HP, Hypothetical Protein. Nt_HK: HK with an «orange» N-terminal domain exhibiting similarities with cyanobacterial KaiB protein and kaiB-like N-terminal KaiC-interacting sensory HK SasA (see text). Orange branches represent *kaiC* genes (ST or SS) localized in the vicinity of a *kaiB* gene. Deep-Blue branches represent *kaiC* genes (SY, TY, SF) localized upstream a conserved specific histidine kinase designated Nt_HK (see above and text). Light-blue branches represent *kaiC* genes (SY, NY, SP, SH) branched with deep-blue family, but included in the «third black family» (see text). Black branches represent *kaiC* genes with poorly conserved phosphorylation sites (SS, SA, SF, AF, DY, SL etc…) and more heterogeneous organization. α, β, γ, δ, ε represent α-, β-, γ-, δ-, and ε-Proteobacteria. KaiC^1a^, KaiC^2a^, KaiC^3a^ indicate that the strain «a» contains 3 differents KaiC copies called 1a, 2a and 3a.
(TIFF)

Figure S7 **Sequence alignment of KaiC proteins centered on** ***Syn*** **KaiC phosphorylable residues (T426, S431 and T432).** Conserved T, ST are red coloured, S replacing T are green coloured, Y replacing T are blue coloured, T replacing S are pink coloured and other replacement with a non phosphorylable residue are italicized. KaiC proteins exhibiting one or several replacements with a non phosphorylable residue are in bold. *Rta*KaiC are underlined. Cyano: Cyanobacteria; α, β, γ, δ, ε represent α-, β-, γ-, δ-, and ε-Proteobacteria.
(TIFF)

Table S1 **Genes involved in autotrophic dicarboxylate/hydroxybutyrate cycle (carbohydrate metabolism), energetic metabolism, dissimilative nitrate reduction and cofactors and vitamins synthesis in** ***Ramlibacter tataouinensis*** **TTB310.** Note that: i) ATP is generated by classical and complete oxidative phosphorylation including the five complexes [complex I (NADH ubiquinone oxidoreductase), complex II (fumarate reductase/succinate dehydrogenase), complex III (cytochrome bc1), complex IV (cytochrome oxidase), and complex V], plus two additional oxidases [one additional cytochrome oxidase, and one cytochrome d (bd-I) ubiquinone oxidase, known to function at low oxygen concentration in *Escherichia coli*]. ii) enzymes for complete denitrification and dinitrogen reduction are absent, and iii) key enzymes for the biosynthesis of thiamine, pantothenate and biotin are missing, confirming the growth factor requirement of this bacterium [4].
(XLS)

Table S2 **Carbohydrate-active enzymes (CAZymes) found in** ***Ramlibacter tataouinensis*** **TTB310.** Note that this table contains: i) a list of CAZymes found in strain TTB310, ii) a comparison of CAZymes from strain TTB310 against seven betaproteobacterial genomes, and iii) a list of exported and potentially secreted glycosyl hydrolases.
(XLS)

Table S3 **Fatty acid composition of each membrane glycerolipid class extracted from** ***Ramlibacter tataouinensis*** **TTB310 cells.** PE, phosphatidylethanolamine, PC, phosphatidylcholine, PG, phosphatidylglycerol, DPG, diphosphatidylglycerol, PI, phosphoinositol, FA, fatty acid.
(DOC)

Table S4 **Characteristics of cell envelope transport systems in** ***Ramlibacter tataouinensis*** **TTB310.** This table contains genes involved in: general export pathway (Sec translocation, SRP insertion and Tat translocation pathways, including predicted Tat substrates), Outer Membrane Protein (OMP) insertion machinery, Outer Membrane (OM) lipoproteins synthesis (including Predicted lipoproteins), type I, type II and type III secretion systems, and type IV pili machinery.
(XLS)

Table S5 **Additional signal transduction, regulator and bifunctional proteins in** ***Ramlibacter tataouinensis*** **TTB310.**
(DOC)

Text S1 **Experimental conditions for live optical imaging of** ***Ramlibacter tataouinensis*** **TTB310 (video S1).**
(DOC)

Video S1 **Live optical imaging of** ***Ramlibacter tataouinensis*** **TTB310 exhibiting both “cyst-to-rod” division step (in the middle of the screen) and “** ***rod-rod*** **” division (last images) (See Text S1 for details).**
(AVI)
