# Supplementary figures and images for: The Cyst-Dividing Bacterium Ramlibacter tataouinensis TTB310 Genome Reveals a Well-Stocked Toolbox for Adaptation to a Desert Environment
Source: PLoS One. 2011 Sep 1;6(9):e23784. doi: 10.1371/journal.pone.0023784 (PMC3164672; doi:10.1371/journal.pone.0023784)

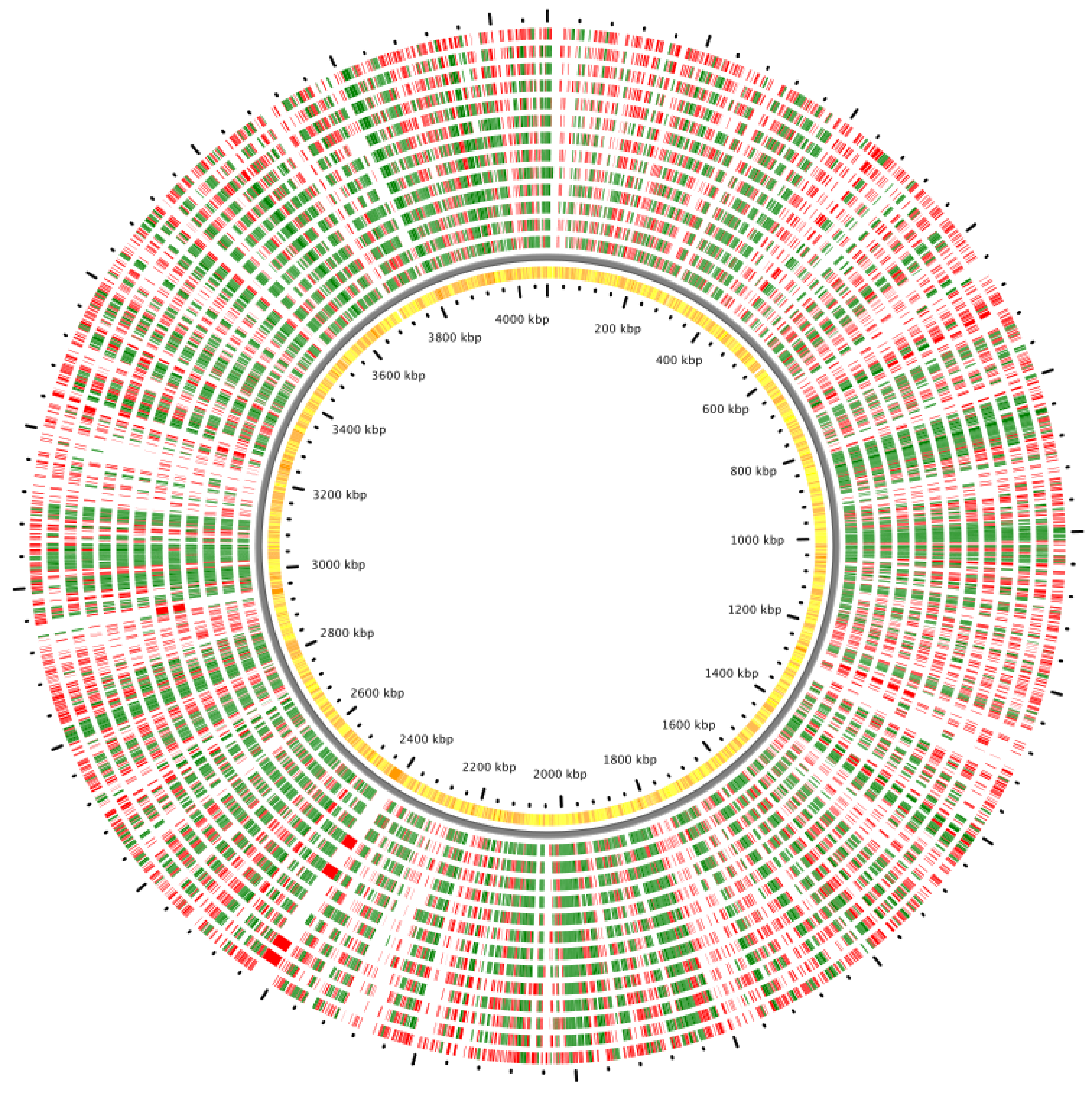

Supplement: Figure S1 — Comparison of the Ramlibacter tataouinensis TTB310 genome against the closest proteobacterial genomes. Similarity searches were carried out between strain TTB310 and all the complete proteomes present in NCBI database, using BLASTP. The figure was generated with the results of the thirteen most similar genomes (12 betaproteobacteria, 1 alphaproteobacterium). Genomes are represented by successive circles made of coloured sticks representing individual genes. Color code of sticks: orange, strain TTB310 CDS forward; yellow: strain TTB310 CDS reverse; green: similar genes present and found in the same genomic environment in the other genomes (synteny); red: similar genes present in the other genomes. White holes represent an absence of similar genes in the other genomes. Names of the thirteen strains used for genome comparison classified from the inner (most similar) to the outside of the circle: Polaromonas sp. JS666, Delftia acidovorans SPH-1, Acidovorax avenae subsp. citrulli AAC00-1, Polaromonas naphthalenivorans CJ2, Acidovorax sp. JS42, Leptothrix cholodnii SP-6, Methylibium petroleiphilum PM1, Rhodoferax ferrireducens T118, Azoarcus sp. BH72, Ralstonia eutropha H16 chromosome 1, Bordetella petrii DSM 12804, Burkholderia xenovorans LB400 chromosome 1, Bradyrhizobium sp. ORS278. (TIFF) [file pone.0023784.s001.tiff]

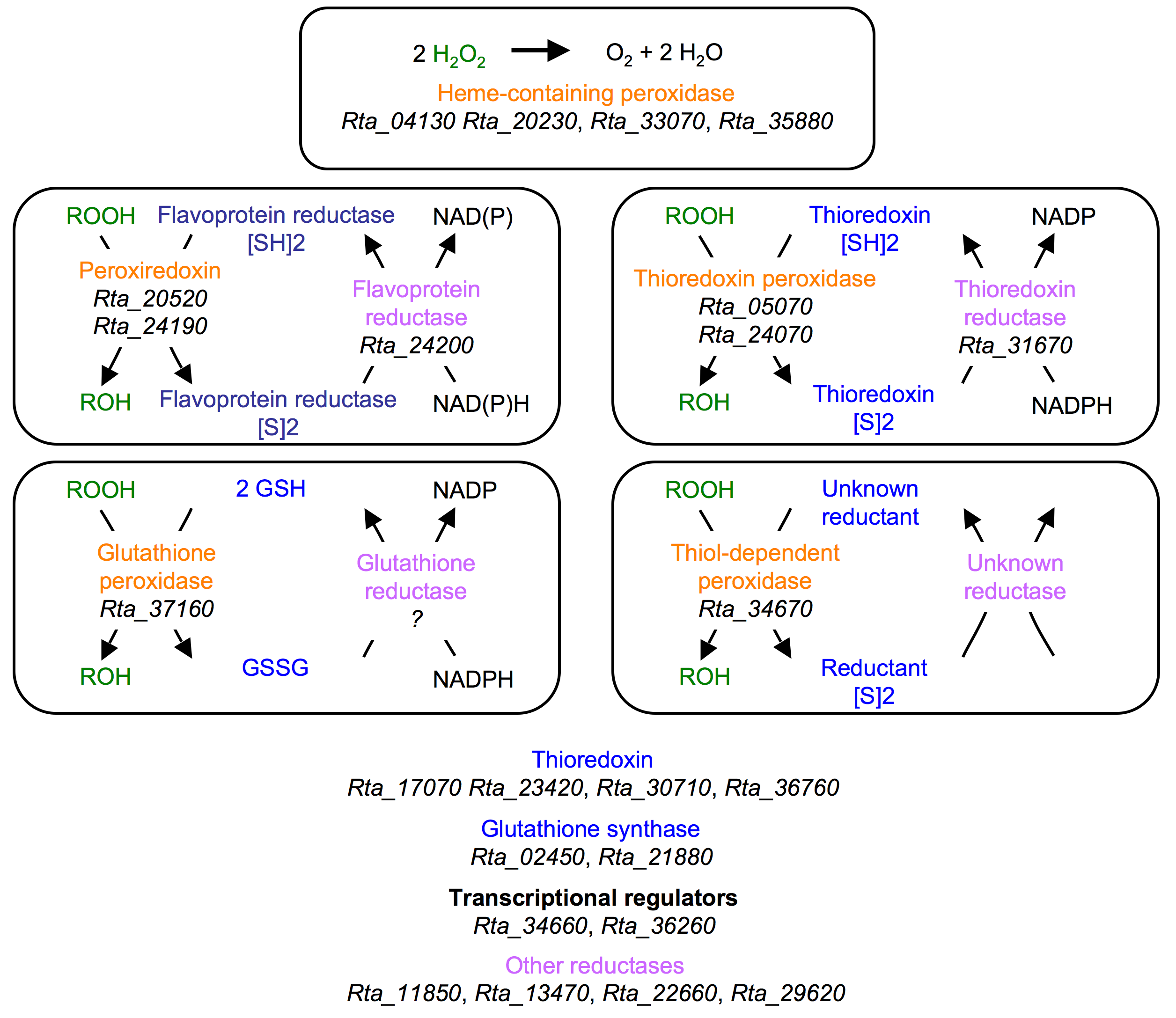

Supplement: Figure S2 — Genes of Ramlibacter tataouinensis TTB310 potentially involved in peroxide scavenging pathways. (TIFF) [file pone.0023784.s002.tiff]

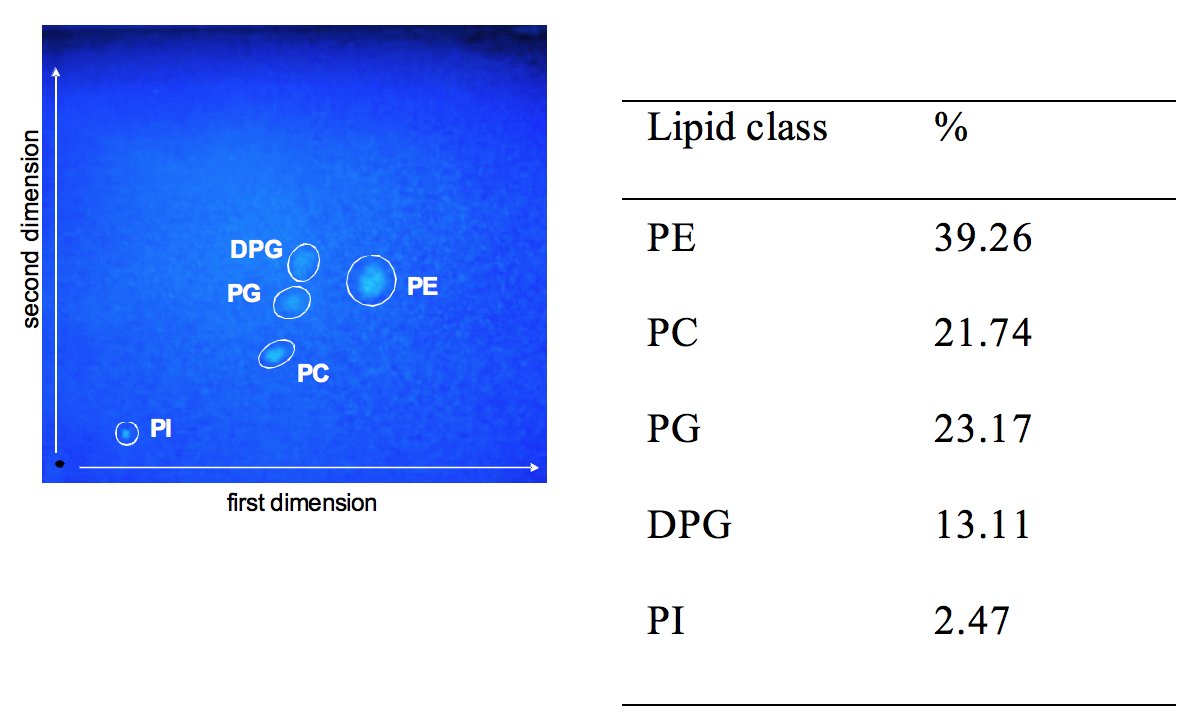

Supplement: Figure S3 — Glycerolipid composition of Ramlibacter tataouinensis TTB310 membranes. PE, phosphatidylethanolamine, PC, phosphatidylcholine, PG, phosphatidylglycerol, DPG, diphosphatidylglycerol, PI, phosphoinositides. Glycerolipids (100 µg) were resolved by two-dimensional thin layer chromatography (first dimension, chloroform/methanol/water 65∶25∶4; second dimension, chloroform/acetone/methanol/acetic acid/water 100∶40∶20∶10) and visualized after 8-anilino-1-naphthalenesulfonic acid spray. (TIFF) [file pone.0023784.s003.tiff]

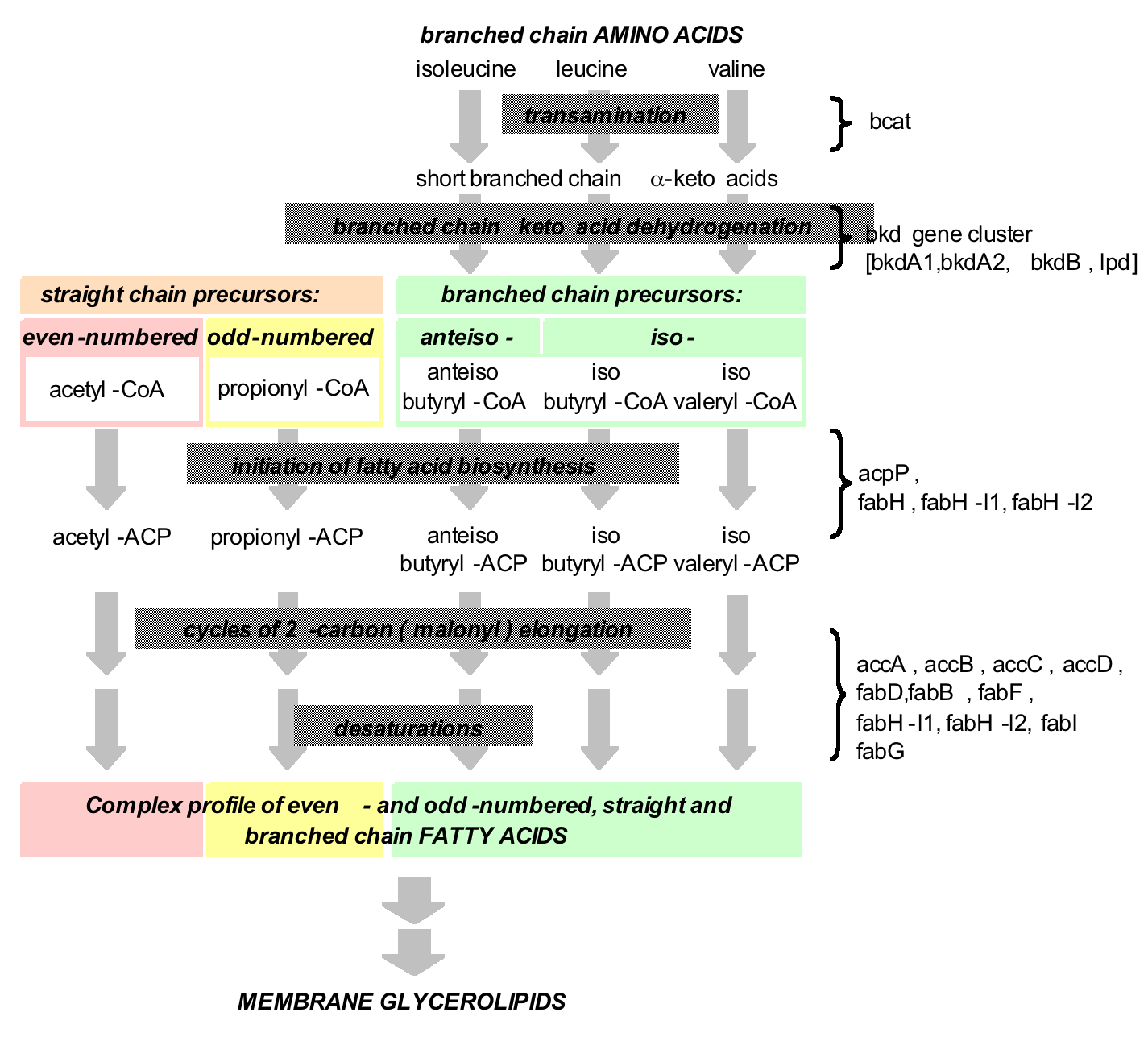

Supplement: Figure S4 — Biosynthesis of even- and odd-numbered, straight and branched chain fatty acids from acetyl-CoA, propionyl-CoA and branched chain amino acids derivatives as starting units in Ramlibacter tataouinensis TTB310. Determining steps for the distribution of fatty acid molecular species in the final profile include the branched chain amino acid transaminase (bcaT), the α-keto acid dehydrogenase (bkd) cluster and the β-ketoacyl-ACP synthase III (fabH). (TIFF) [file pone.0023784.s004.tiff]

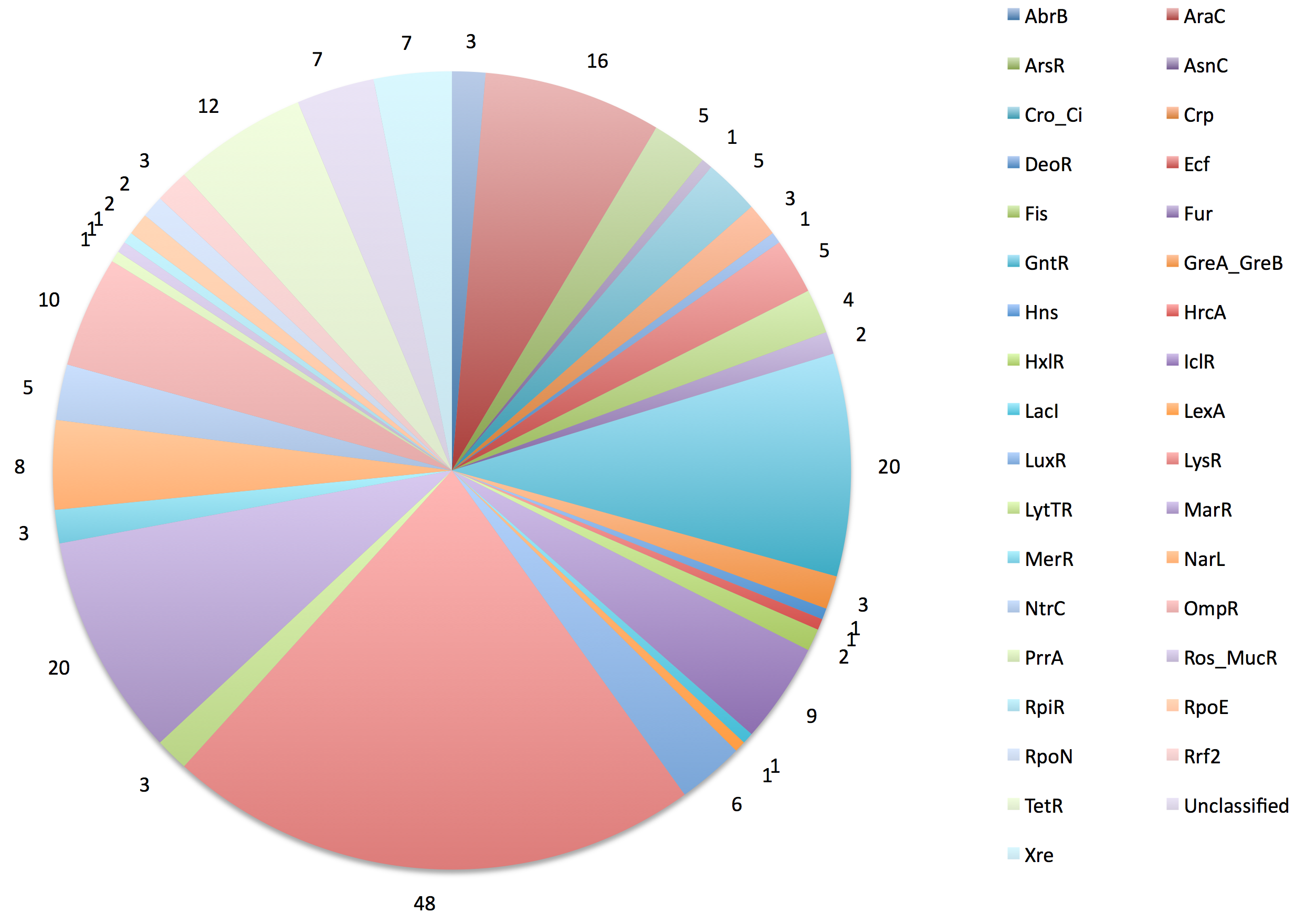

Supplement: Figure S5 — DNA-binding proteins in Ramlibacter tataouinensis TTB310. This figure represents the distribution of the transcription factors found in R. tataouinensis. (TIFF) [file pone.0023784.s005.tiff]

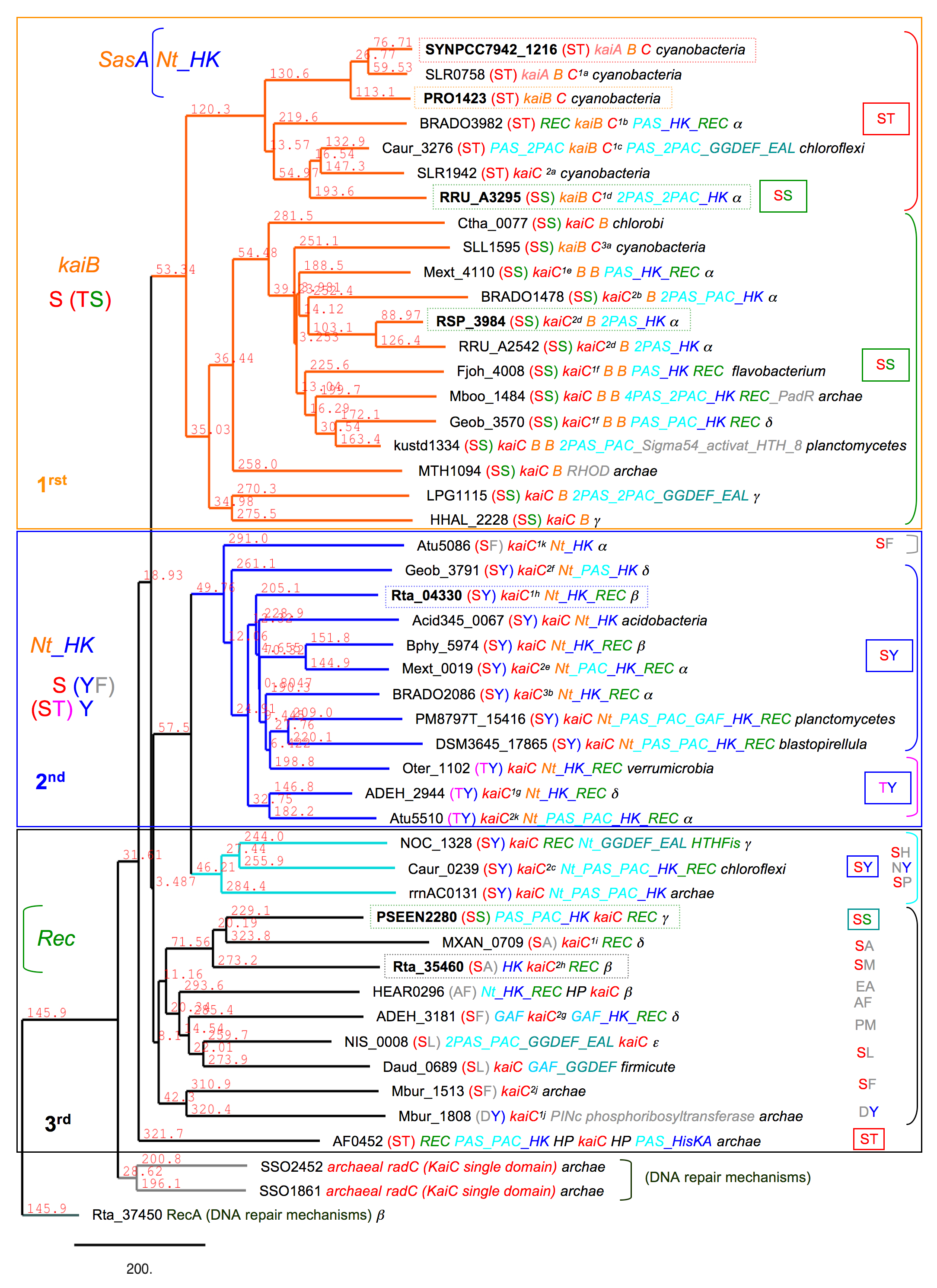

Supplement: Figure S6 — Representation of prokaryotic predicted KaiC proteins according to (1) their TULIP tree position, (2) nature of their phosphorylable sites and (3) their genetic organization. Proteins were classified using a distance matrix derived from Z-value probabilities (see Materials and Methods). We have integrated the RecA protein (Rta_37450, 351 residues) as an outgroup and two archaeal KaiC single domain proteins (SSO1861, 280 residues; SSO2452, 262 residues) recently classified as archaeal RadC and thought to be implicated in DNA repair [77]. (ST) represent the nature of the conserved KaiC phosphorylation sites residues (S, serine; T, threonine; Y, tyrosine; F, phenylalanine; A, alanine; L, leucine; H, histidine; D, aspartic acid). KaiC neighbouring proteins were represented according to their protein domain contents: REC, single domain receiver protein; Nt_PAS_PAC_GAF_HK_REC, hybrid histidine kinase with N-terminal domain composed of a N-terminal region, one PAS, one PAS and one GAF domains; PAS_2PAC_GGDEF_EAL, protein containing one PAS, two PAC, one GGDEF and one EAL domains. HP, Hypothetical Protein. Nt_HK: HK with an «orange» N-terminal domain exhibiting similarities with cyanobacterial KaiB protein and kaiB-like N-terminal KaiC-interacting sensory HK SasA (see text). Orange branches represent kaiC genes (ST or SS) localized in the vicinity of a kaiB gene. Deep-Blue branches represent kaiC genes (SY, TY, SF) localized upstream a conserved specific histidine kinase designated Nt_HK (see above and text). Light-blue branches represent kaiC genes (SY, NY, SP, SH) branched with deep-blue family, but included in the «third black family» (see text). Black branches represent kaiC genes with poorly conserved phosphorylation sites (SS, SA, SF, AF, DY, SL etc…) and more heterogeneous organization. α, β, γ, δ, ε represent α-, β-, γ-, δ-, and ε-Proteobacteria. KaiC1a, KaiC2a, KaiC3a indicate that the strain «a» contains 3 differents KaiC copies called 1a, 2a and 3a. (TIFF) [file pone.0023784.s006.tiff]

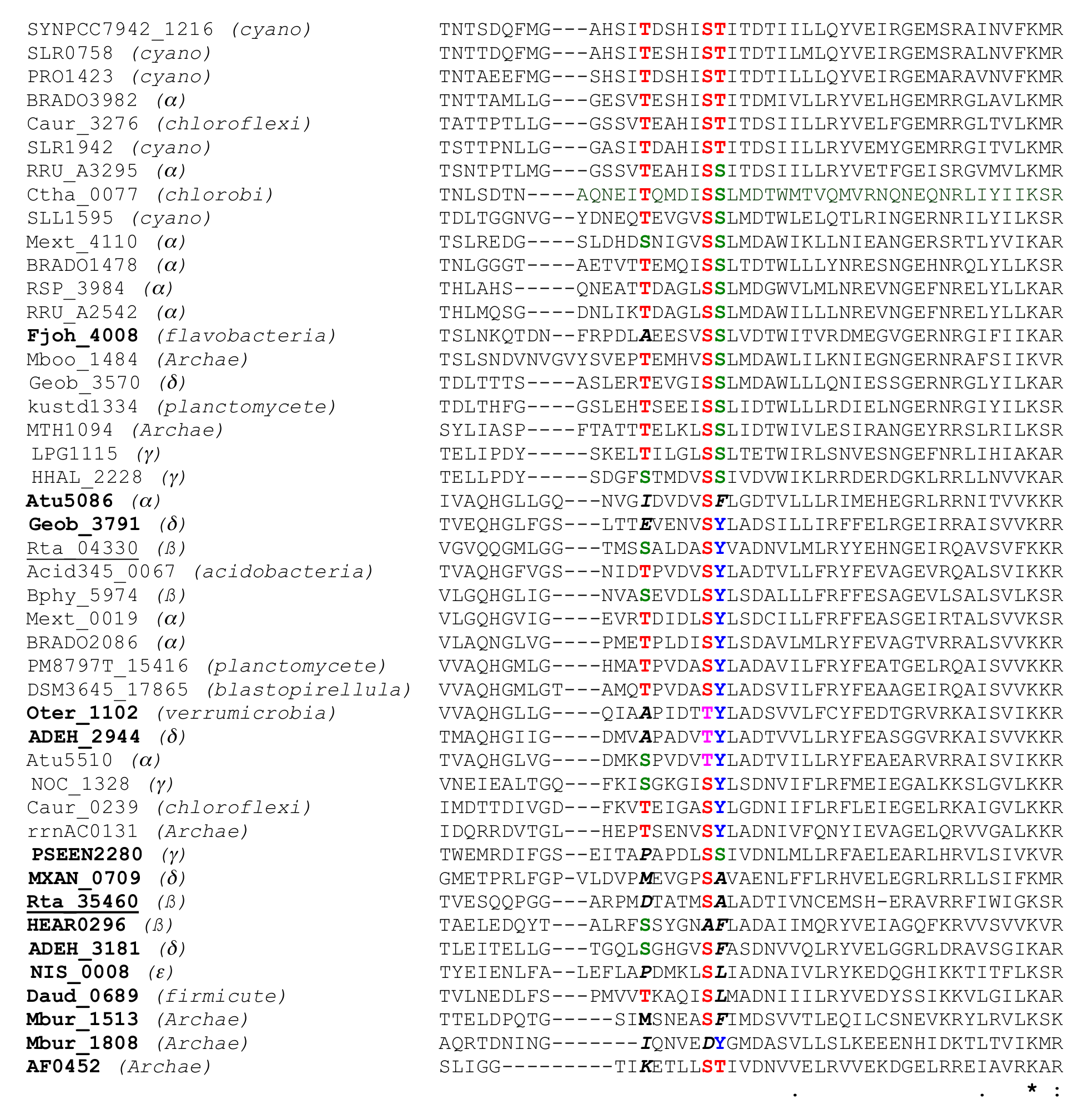

Supplement: Figure S7 — Sequence alignment of KaiC proteins centered on Syn KaiC phosphorylable residues (T426, S431 and T432). Conserved T, ST are red coloured, S replacing T are green coloured, Y replacing T are blue coloured, T replacing S are pink coloured and other replacement with a non phosphorylable residue are italicized. KaiC proteins exhibiting one or several replacements with a non phosphorylable residue are in bold. RtaKaiC are underlined. Cyano: Cyanobacteria; α, β, γ, δ, ε represent α-, β-, γ-, δ-, and ε-Proteobacteria. (TIFF) [file pone.0023784.s007.tiff]
