# Supplementary material for: The Cyst-Dividing Bacterium Ramlibacter tataouinensis TTB310 Genome Reveals a Well-Stocked Toolbox for Adaptation to a Desert Environment
Source: PLoS One. 2011 Sep 1;6(9):e23784. doi: 10.1371/journal.pone.0023784 (PMC3164672; doi:10.1371/journal.pone.0023784)
Supplement: Table S3 — Fatty acid composition of each membrane glycerolipid class extracted from Ramlibacter tataouinensis TTB310 cells. PE, phosphatidylethanolamine, PC, phosphatidylcholine, PG, phosphatidylglycerol, DPG, diphosphatidylglycerol, PI, phosphoinositol, FA, fatty acid. (DOC) [file pone.0023784.s010.doc]

**Table S3. Fatty acid composition of each membrane glycerolipid class extracted from *Ramlibacter tataouinensis* TTB310 cells.** PE, phosphatidylethanolamine, PC, phosphatidylcholine, PG, phosphatidylglycerol, DPG, diphosphatidylglycerol, PI, phosphoinositol, FA, fatty acid.

| **Fatty acid** | **PE** | **PC** | **PG** | **DPG** | **PI** | **Total glycerolipid** |
| --- | --- | --- | --- | --- | --- | --- |
| C14:0 | 0.24 | 0.21 | nd | 0.46 | 0.51 | 0.22 |
| C14:0-*iso* | nd | 0.20 | nd | 0.81 | nd | 0.15 |
| C15:0 | 3.04 | 2.71 | 0.76 | 2.41 | 1.49 | 2.32 |
| C15:0-*anteiso* | 0.17 | 0.54 | nd | 0.98 | 1.71 | 0.36 |
| C15:0-*iso* | nd | 0.59 | nd | 0.93 | nd | 0.25 |
| C16:0 | 19.80 | 21.46 | 9.60 | 14.19 | 6.80 | 16.73 |
| C16:0-*iso* | 0.64 | 0.23 | nd | 0.94 | 1.66 | 0.47 |
| C16:1 | 3.84 | 0.22 | 0.93 | 3.66 | 0.69 | 2.27 |
| C17:0 | 14.99 | 14.87 | 9.92 | 9.51 | 0.51 | 12.71 |
| C17:0-*anteiso* | 1.39 | 1.01 | nd | 0.97 | nd | 0.90 |
| C17:0-*iso* | 1.32 | nd | nd | 1.50 | nd | 0.72 |
| C17:1 | 9.36 | 6.67 | 5.19 | 5.98 | 0.23 | 7.14 |
| C18:0 | 1.62 | 1.94 | 1.45 | 2.26 | 2.40 | 1.75 |
| C18:0-*iso* | 2.90 | 3.31 | nd | 1.79 | nd | 2.10 |
| C18:1 | 12.46 | 12.90 | 6.22 | 8.25 | 1.43 | 10.28 |
| C18:2 | 10.55 | 11.51 | 6.34 | 9.85 | 6.34 | 9.58 |
| C18:3 | 4.16 | 5.24 | 3.05 | 2.89 | 1.37 | 3.90 |
| C20:0 | 0.27 | 0.58 | 0.68 | 0.42 | 1.77 | 0.49 |
| C20:1 | nd | nd | nd | 1.42 | 2.80 | 0.26 |
| C20:>1 | 2.08 | 0.71 | 0.76 | 0.95 | 4.17 | 1.38 |
| C22:0 | 0.11 | 0.21 | 16.97 | 1.02 | 0.86 | 4.19 |
| C22:1 | 0.70 | 1.69 | 1.31 | 1.40 | 6.00 | 1.28 |
| C22:2 | 9.19 | 9.83 | 0.41 | 20.34 | 47.60 | 9.71 |
| C22:>2 | nd | 1.33 | nd | 0.71 | 3.66 | 0.47 |
| C24:0 | nd | nd | 35.44 | nd | nd | 8.23 |
| undetermined | 1.16 | 2.01 | 0.99 | 6.36 | 8.00 | 2.16 |
| Total | 100 | 100 | 100 | 100 | 100 | 100 |
|  |  |  |  |  |  |  |
| Odd | 30.27 | 26.38 | 15.87 | 22.28 | 3.94 | 24.40 |
| -*anteiso* | 2.88 | 2.92 | nd | 1.95 | 1.71 | 1.26 |
| *-iso* | 1.32 | 0.59 | nd | 1.50 | nd | 0.97 |
| Even | 69.73 | 73.62 | 84.13 | 77.72 | 96.06 | 75.60 |
| -*iso* | 3.54 | 3.74 | nd | 3.54 | 1.66 | 4.28 |
| Branched-chain FA | 7.74 | 7.25 | nd | 6.99 | 3.37 | 6.51 |
| Unsaturated FA | 52.34 | 50.1 | 24.21 | 55.45 | 74.29 | 46.27 |
| Very long chain FA | 12.35 | 14.35 | 55.57 | 26.44 | 67.39 | 26.01 |
