# Supplementary material for: The Cyst-Dividing Bacterium Ramlibacter tataouinensis TTB310 Genome Reveals a Well-Stocked Toolbox for Adaptation to a Desert Environment
Source: PLoS One. 2011 Sep 1;6(9):e23784. doi: 10.1371/journal.pone.0023784 (PMC3164672; doi:10.1371/journal.pone.0023784)
Supplement: Table S5 — Additional signal transduction, regulator and bifunctional proteins in Ramlibacter tataouinensis TTB310. (DOC) [file pone.0023784.s012.doc]

**Table S5. Additional signal transduction, regulator and bifunctional proteins in *Ramlibacter tataouinensis* TTB310**.

| **CDS *Rta*** | **Description** |
| --- | --- |
| *Rta_03040* | Related to signal transduction protein: sensor, cNMP-binding domain |
| *Rta_09840* | Related to regulator protein: GGDEF domain |
| *Rta_10530* | Related to signal transduction protein: sensor, GAF domain |
| *Rta_13360* | Related to signal transduction protein: sensor, GAF domain; transcriptional regulator, Fis family |
| *Rta_13470* | related to bifunctional protein: sensor, cNMP-binding domain; thioredoxin reductase |
| *Rta_14800* | Related to signal transduction protein: sensor, PAS/PAC domains; transcriptional regulator, LuxR family |
| *Rta_15610* | Related to signal transduction protein: sensor, PAS domain; Sigma-54 interaction domain; transcriptional regulator, Fis family |
| *Rta_18750* | Related to regulator protein: GGDEF domain |
| *Rta_21140* | Related to signal transduction protein: sensor, PAS/PAC domains |
| *Rta_22200 (rsbR)* | Candidate signal transduction protein (modulator protein RsbR): STAS domain |
| *Rta_22210 (rsbS)* | Candidate signal transduction protein (antagonist protein RsbS): STAS domain |
| *Rta_22220 (RsbT)* | Candidate signal transduction protein (RsbT): serine/threonine protein kinase (GHKL ATPase domain) |
| *Rta_22230* | Related to signal transduction protein, fusion protein: serine/threonine protein kinase (GHKL ATPase and PP2C domains) |
| *Rta_25630* | Related to regulator protein (GGDEF domain), membrane protein |
| *Rta_33020* | Related to signal transduction protein: sensor, PAS/PAC domains; regulator, GGDEF/EAL domains |
| *Rta_33040* | Related to signal transduction protein: sensor, GAF domain; regulator, GGDEF/EAL domains |
| *Rta_37350* | Related to bifunctional protein: sensor, GAF domain; type IV pilus assembly protein PilB, putative traffic ATPase |
| *Rta_38190* | Related to signal transduction protein: sensor, cNMP-binding domain |
