# Supplementary material for: The Cyst-Dividing Bacterium Ramlibacter tataouinensis TTB310 Genome Reveals a Well-Stocked Toolbox for Adaptation to a Desert Environment
Source: PLoS One. 2011 Sep 1;6(9):e23784. doi: 10.1371/journal.pone.0023784 (PMC3164672; doi:10.1371/journal.pone.0023784)
Supplement: Text S1 — Experimental conditions for live optical imaging of Ramlibacter tataouinensis TTB310 (video S1). (DOC) [file pone.0023784.s013.doc]

**Text S1. Experimental conditions for live optical imaging of *Ramlibacter tataouinensis* TTB310 (video S1)**

**Single cell imaging**

For live optical imaging of strain TTB310 cells, we used a high magnification optical microscope with a 100X phase contrast objective (Zeiss Axiovert 200 with an oil contact lens Plan-Neofluar100xPh3 NA=1.3 and a supplementary 1.6 Optovar lens). For detection we used a back-illuminated CCD system, with 1340x1300 pixels each of 20 µm x 20 µm size, cooled to –100 °C (Roper Scientific‑Princeton Instruments Camera, CT 1300B Cryotiger). The data acquisition was entirely automated with custom-made software, interfaced with the camera control software, WinView/32. For automatic focusing we used phase contrast imaging (exposure 200 ms under low intensity bright-field light). One field of view preceded by an automatic focusing was taken every 20 min, and the length of the movie shown here is approximately 26 h.

**Cells, growth chamber**

The strain TTB310 cells were grown in 1/10 TBS medium at 30 °C overnight in dark. The growth chambers for the microscope used were on 50x9 mm Petri dishes, with a N°0 glass coverslip (MatTek Corporation P50G-0-14F) glued inside. 0.5 µl of cells was spotted on the coverslip, coated with 100 µl of liquid 6 % SeaPlaque low-melt agarose in media and immediately covered and insulated with a transparent 0.4 µm pore size PET track-etched membrane (Falcon) and paraffin sealed. The Petri dish was then filled with 30 °C media, closed, and installed under the microscope. In this way, movement of the cells was highly restrained by the agarose gel, but still with a homogeneous access for all cells to growth media and light, while a sufficiently transparent light pathway allowed phase contrast microscopy.
